# Change in *N*-Glycosylation of Plasma Proteins in Japanese Semisupercentenarians

**DOI:** 10.1371/journal.pone.0142645

**Published:** 2015-11-11

**Authors:** Yuri Miura, Noritaka Hashii, Hiroki Tsumoto, Daisuke Takakura, Yuki Ohta, Yukiko Abe, Yasumichi Arai, Nana Kawasaki, Nobuyoshi Hirose, Tamao Endo, SONIC (Septuagenarians, Octogenarians, Nonagenarians Investigation with Centenarians)

**Affiliations:** 1 Research Team for Mechanism of Aging, Tokyo Metropolitan Institute of Gerontology, Tokyo, Japan; 2 Division of Biological Chemistry and Biologicals, National Institute of Health Sciences, Tokyo, Japan; 3 Center for Supercentenarian Research, Keio University School of Medicine, Tokyo, Japan; Pacific Northwest National Laboratory, UNITED STATES

## Abstract

An *N*-glycomic analysis of plasma proteins was performed in Japanese semisupercentenarians (SSCs) (mean 106.7 years), aged controls (mean 71.6 years), and young controls (mean 30.2 years) by liquid chromatography/mass spectrometry (LC/MS) using a graphitized carbon column. Characteristic *N*-glycans in SSCs were discriminated using a multivariate analysis; orthogonal projections to latent structures (O-PLS). The results obtained showed that multi-branched and highly sialylated *N*-glycans as well as agalacto- and/or bisecting *N*-glycans were increased in SSCs, while biantennary *N*-glycans were decreased. Since multi-branched and highly sialylated *N*-glycans have been implicated in anti-inflammatory activities, these changes may play a role in the enhanced chronic inflammation observed in SSCs. The levels of inflammatory proteins, such as CRP, adiponectin, IL-6, and TNF-α, were elevated in SSCs. These results suggested that responses to inflammation may play an important role in extreme longevity and healthy aging in humans. This is the first study to show that the *N*-glycans of plasma proteins were associated with extreme longevity and healthy aging in humans.

## Introduction

Aging is not caused by a single factor or process; it is modulated by various genetic and environmental factors such as oxidative stress and lifestyle. However, the mechanisms underlying the aging process currently remain unclear [1]. Semisupercentenarians (SSCs; older than 105 years) are regarded as a model of human longevity because they have aged successfully [2]. Therefore, analyses of SSCs are expected to assist in elucidating the human aging process. We have physiologically and biochemically analyzed SSCs, and demonstrated that the content of oxidative stress-related proteins in the plasma of SSCs was altered from a young age [3]. These findings suggest that the regulatory mechanisms underlying oxidative stress are important for human longevity and healthy aging.

Since post-translational modifications to proteins do not occur according to a genetic template, they are sensitive to various biological changes. Typical modifications to proteins, such as phosphorylation, glycosylation, and acetylation, depend on the activities of enzymes such as kinases, glycosyltransferases, and acetylases, respectively. Since the aging process is modulated by environmental factors, e.g. oxidative stress, heat shock, etc [1], a focus on age-dependent alterations in post-translational modifications in SSCs is considered important for obtaining a deeper understanding of human longevity and healthy aging.

Glycosylation is one of the most common post-translational modifications to proteins. Recent studies suggest that glycans modify the functions of proteins and play important roles in various biological processes such as molecular recognition, cell adhesion, and immunological defense systems. The biosynthesis of glycans is known to depend on the activities of glycosyltransferases (transfer of sugars) and glycosidases (hydrolysis of sugars), and their activities are easily altered by changes in the physiological conditions of cells. Therefore, although glycans are highly susceptible to changes in the biological environment such as those caused by various disorders [4–7] and aging [8], the mechanisms responsible have not yet been examined in detail. *N*-linked glycosylation (*N*-glycan), which is covalently conjugated to asparagine residues in a consensus sequence (Asn-X-Ser/Thr), is synthesized in the endoplasmic reticulum, in which a lipid-linked precursor oligosaccharide is attached to a protein, followed by the concerted action of glycosyltransferases in the Golgi apparatus. More than half of mammalian proteins are estimated to be glycosylated. Human plasma proteins, except for albumin and CRP (C-reactive protein), are mostly modified by glycans. Furthermore, since plasma proteins are derived from various tissues and organs, their properties are affected by the physiological or pathological conditions of various tissues and organs, indicating that plasma proteins and their glycans are good targets for monitoring healthy conditions.

*N*-glycan structures have a number of positional isomers and anomeric configurations including branching; therefore, they are very diverse. As a result, difficulties have been associated with obtaining *N*-glycan structural information until recently. Among several analytical methods, mass spectrometry (MS) is currently the most efficient and promising analytical tool for elucidating *N*-glycan structures. *N*-Glycan analyses have often been performed using liquid chromatography (LC) and capillary electrophoresis in combination with MS [9, 10]. These methods are well established and regarded as platform technologies for *N*-glycan profiling; however, manually distinguishing complex and unclear differences between the *N*-glycan heterogeneities of samples when significant quantitative and qualitative changes are not observed is very challenging. We applied previously orthogonal projections to latent structures (O-PLS) in order to characterize the *N*-glycan heterogeneities of erythropoietin using the peak area ratios of *N*-glycans in mass spectra obtained by LC/MS [11]. Differences in *N*-glycan heterogeneities were visualized and digitalized using these methods, and characteristic *N*-glycans were successfully identified. Previous studies reported the application of O-PLS to the search for biomarkers in proteomics [12–15].

In the present study, we performed a glycomic analysis of *N*-glycan in the plasma proteins of SSCs using LC/MS and O-PLS, and demonstrated the characteristic structures of *N*-glycans in SSCs, which may play a role in extreme longevity and successful aging in humans.

## Materials and Methods

### Subjects

Six female semisupercentenarians (SSCs, mean age 106.7 ± 0.5 years) were recruited in this study. None were in an acute care situation or receiving tube feeding. The disease histories of these SSCs included coronary artery disease, stroke, diabetes, hypertension, and cancer. Five female aged subjects (mean age 71.6 ± 1.5 years) and 5 female young subjects (mean age 30.2 ± 8.1 years) were recruited as healthy volunteers. The young subjects were free from diseases and had no relevant history, such as coronary artery disease, stroke, diabetes, hypertension, and cancer. The aged subjects were also free from diseases, but had histories of coronary artery disease and hypertension. All subjects enrolled in this study were Japanese. Twenty milliliters of non-fasting venous blood was collected, and plasma was immediately separated by centrifugation at 4°C and stored at -80°C until subsequent assays. This study was approved by the Ethics Committees of the Tokyo Metropolitan Institute of Gerontology (approval number 1). All participants in this study gave written informed consent to participate.

### Materials

*N*-Glycosidase F was purchased from Roche Diagnostics (Mannheim, Germany). Guanidine hydrochloride was purchased from Nacalai Tesque (Kyoto, Japan). Dithiothreitol and monoiodoacetate were purchased from Sigma (St. Louis, MO, USA). All other reagents were of the highest quality available.

### Sample Preparation for *N*-glycan Profile Analyses

Plasma (3 μl) was dissolved in 8 M guanidine hydrochloride / Tris-HCl (pH 8.6). The mixture was reduced by the addition of dithiothreitol for 30 min at 65°C, followed by alkylation with sodium monoiodoacetate for 40 min at room temperature in the dark. The resulting mixture was applied to a PD-10 column (GE Healthcare, Little Chalfont, UK) to remove the reagents, and a fraction of the carboxymethylated proteins was dried. The sample was re-dissolved in 50 mM sodium phosphate buffer containing 10 mM EDTA (pH 8.0). After protein concentration was determined using a protein assay kit (Bio-Rad Laboratories, Hercules, CA, USA), 5 U of peptide *N*-glycosidase F, which cleaves between the innermost GlcNAc and asparagine residues of *N*-linked glycoproteins, was added to the sample (100 μg protein) and incubated for 16 h at 37°C. Proteins were removed by precipitation with ice cold methanol (60%) and centrifugation (4°C, 8,000 g x 5 min), and the supernatant including *N*-glycan was evaporated. *N*-glycans were reduced in 500 μl of 0.5 M sodium borohydride at room temperature for 16 h and neutralized with acetic acid. Reduced *N*-glycans were recovered with a solid-phase extraction cartridge (EnviCarb C, Supelco, Bellefonte, PA, USA), lyophilized, and re-dissolved in 25 μl of ultrapure water.

### Liquid Chromatography/Multiple Stage Mass Spectrometry (LC/MS^n^)

Analyses of sodium borohydride-reduced *N*-glycans were performed using liquid chromatography/multiple-stage mass spectrometry (LC/MS^n^). Chromatographic separation was performed using an UltiMate 3000 RSLCnano LC system (Thermo Fisher Scientific, San Jose, CA, USA) with a graphitized carbon column (Hypercarb, 0.1 x 150 mm, 5 μm; Thermo Fisher Scientific). The mobile phase was 5 mM ammonium bicarbonate containing 2% acetonitrile (buffer A) and 5 mM ammonium bicarbonate containing 80% acetonitrile (buffer B). *N*-glycans were separated at a flow rate of 500 nl/min with a linear gradient of 15%–90% buffer B for 60 min.

A mass spectrometric analysis of *N*-glycans was performed using Fourier transform ion cyclotron resonance linear and ion trap type mass spectrometers (FTMS/ITMS, LTQ-FT, Thermo Fisher Scientific). The analytical conditions were as follows: full mass scan using FTMS (*m/z* 700–2,000) and data-dependent MS/MS, MS/MS/MS, and MS/MS/MS/MS (MS^n^) using ITMS; electrospray voltage in positive and negative ion modes, 2.5 and -2.5 kV, respectively; capillary temperature, 200°C; collision energy for MS^n^ experiments, 35%; maximum injection times for FTMS and MS^n^, 1,250 and 200 ms, respectively; FTMS resolution, 100,000. The peak areas of *N*-glycans were measured using the Thermo Xcalibur 2.2 SP1.48 Qual Browser (Thermo Fisher Scientific).

### Multivariate Analysis (MVA)

The peak area ratios of each *N*-glycan to the total peak area of *N*-glycans were used to perform a principal component analysis (PCA) and orthogonal projections to latent structures (O-PLS) using the MVA software SIMCA-P+ 12.0.1 (Umetrics, Umea, Sweden). Differences in *N*-glycans between generations were visualized in the score plot obtained by PCA, and the characteristic *N*-glycans of each subject were found in the loading plot by O-PLS.

### Contents of *N*-Acetylneuraminic Acid (NeuNAc) in Plasma *N*-Glycans

Plasma samples at the age of 70 years were obtained from SONIC (Septuagenarians, Octogenarians, Nonagenarians Investigation with Centenarians). Samples were prepared from 6 μl plasma according to the same method as the *N*-glycan profile analyses. *N*-Acetylneuraminic acid was released from *N*-glycans by the incubation with *Arthrobacter ureafaciens* sialidase (Nacalai Tesque) in 0.5 M ammonium acetate buffer (pH 5.0) at 37°C for 2 h. The contents of the resulting free *N*-acetylneuraminic acid were measured using a sialic acid (NANA) fluorometric assay kit (BioVision Inc., Milpitas, CA, USA) according to the manufacturer’s instructions, followed by detection and analyses using the EnVision Multilabel Reader (PerkinElmer Inc., Waltham, MA, USA).

### Statistics

A one-way analysis of variance (ANOVA) (IBM SPSS Statistics version 20.0; IBM, Armonk, NY, USA) was used for statistical analyses. Multiple comparisons were performed by the two-sided Dunnett method. Results were considered significant at a p value of < 0.05. In the case of inflammatory parameters such as CRP, adiponectin, IL-6, and TNF-α, data were logarithmically transformed before statistical analyses.

## Results

### Characterization of *N*-Glycan Profiles of Plasma Proteins in SSCs and Aged and Young Controls by the Principal Component Analysis

**Fig 1:**
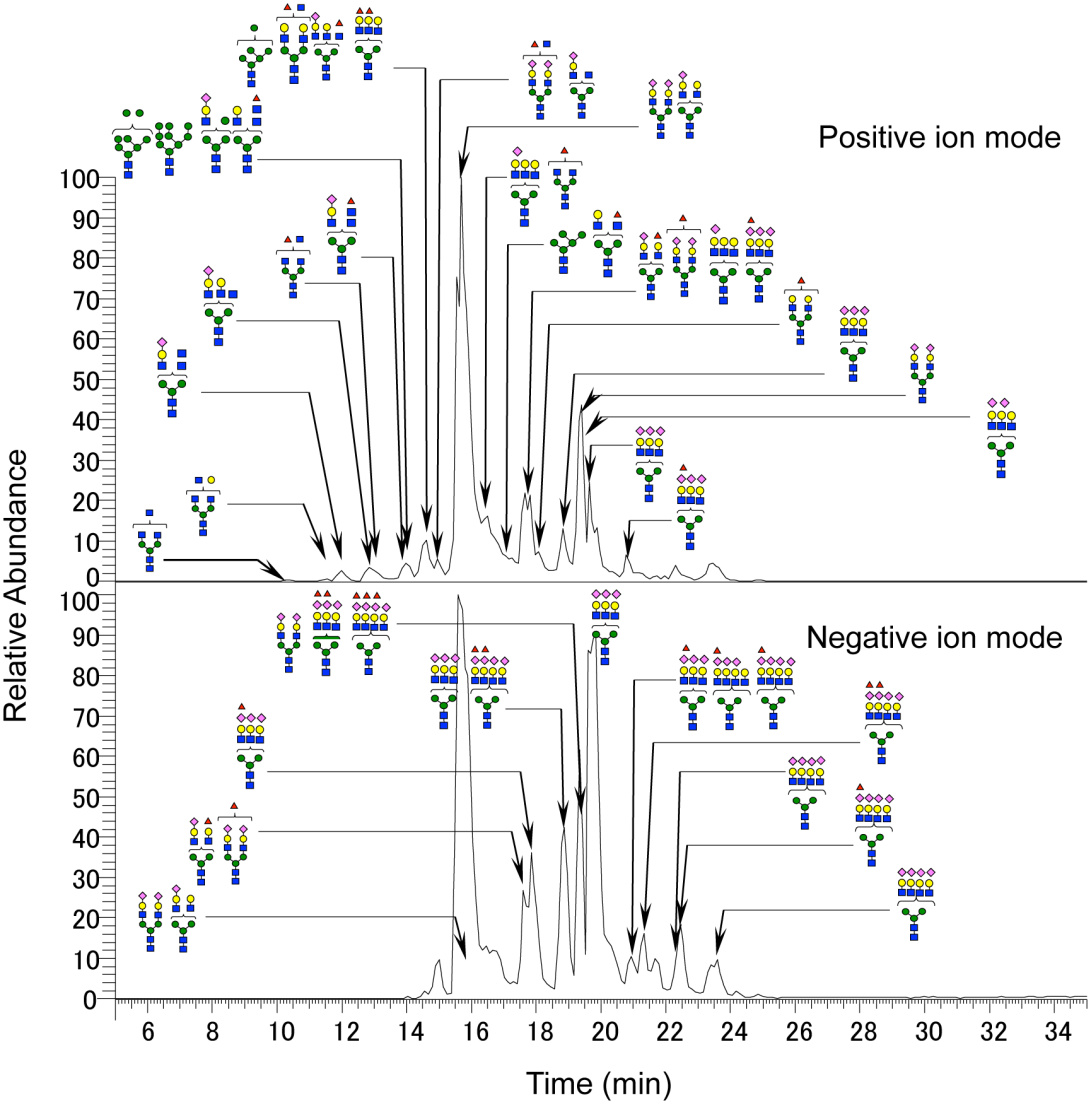
Typical *N*-glycan profile from plasma proteins in SSC. Deduced *N*-glycan structures were added to the base peak chromatogram of the SSC sample. Top and bottom charts represent the positive and negative ion modes, respectively. Vertical axis, relative abundance; horizontal axis, retention time; blue square, *N*-acetylglucosamine; yellow circle, galactose; green circle, mannose; purple diamond, *N*-acetylneuraminic acid; red triangle, fucose.

In order to characterize the *N*-glycans of plasma proteins in SSCs and aged and young controls, we performed *N*-glycan profiling of the three groups. Sodium borohydride-reduced *N*-glycans from each sample were analyzed by LC/MS^n^ in the positive ion mode and negative ion mode. The base peak chromatogram of the reduced *N*-glycans from the plasma samples appeared to be diverse from individuality rather than age groups (S1A and S1B Fig). *N*-glycan structures were deduced on the basis of the accurate mass obtained by FTMS and assignments of fragment ions observed in MS/MS, MS/MS/MS, and MS/MS/MS/MS spectra, resulting in 32 and 18 *N*-glycans containing multiple isomers being identified in the positive and negative ion modes, respectively (Fig 1). The peak areas of precursor ions corresponding to these *N*-glycans were calculated, and the peak area ratios against the total peak area of all *N*-glycans in each ion mode were drawn (S2A and S2B Fig, S1 Table). The distributions of *N*-glycans did not markedly vary among the three groups. The main *N*-glycans identified were disialylated biantennary *N*-glycan (Hex5HexNAc4NeuNAc2) in the positive and negative ion modes, and trisialylated triantennary *N*-glycan (Hex6HexNAc5NeuNAc3) in the negative ion mode.

**Fig 2:**
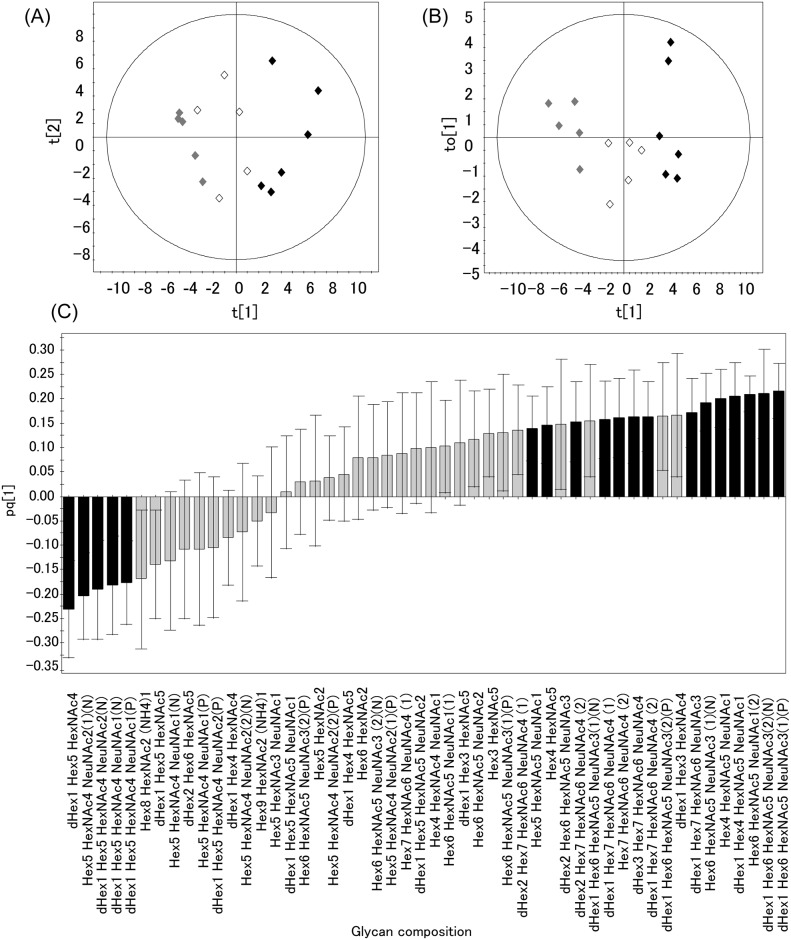
Score plot and loading plot obtained by PCA or O-PLS. (A) A PCA score plot of young controls (shadowed), aged controls (opened), and SSCs (closed) (R2X[1] = 0.329635; R2X[2] = 0.201184; Ellipse: Hotelling T2 (95%)) using the peak area ratios of each *N*-glycan to the total peak area of all identified *N*-glycans. (B) An O-PLS score plot between young controls (shadowed), aged controls (opened), and SSCs (closed). R2X [1] = 0.295051, R2X [XSide Comp. 1] = 0.106066, Ellipse: Hotelling T2 (95%) (C) A loading plot with jack-knifed confidence intervals by O-PLS. The pq[1] value is the weight that combines the X and Y variables. The error bar indicates the standard error (SE) of pq[1] values obtained from 16 samples independently. Glycan compositions were deduced by the accurate mass. Numbers in parentheses indicate isomers. “N” or “P” in parentheses indicates the data obtained from the negative or positive ion mode, respectively. Closed and shadowed columns represent [pq1] / SE > 1.5 and < 1.5, respectively. Row data were summarized in S2 Table. Hex, hexose; HexNAc, *N*-acetylhexosamine; NeuNAc, *N*-acetylneuraminic acid; dHex, deoxyhexose; NH_4_, ammonium.

We performed PCA using peak area ratios in order to visualize differences in *N*-glycan distributions among young controls, aged controls, and SSCs. Three principal components were defined by PCA modeling (R2X [1] = 0.329635, R2X [2] = 0.201184, R2X [3] = 0.106439, Ellipse: Hotelling (T2) 0.95). The score plots for young controls (shadowed symbols), aged controls (open symbols), and SSCs (closed symbols) were shown in Fig 2A. These results demonstrated that young controls, aged controls, and SSCs were roughly clustered into each group, suggesting that *N*-glycans may be characterized by age. Therefore, data were subjected to a discrimination analysis.

### Characterization and Identification of *N*-Glycans by Orthogonal Projections to Latent Structures (O-PLS)

**Fig 3:**
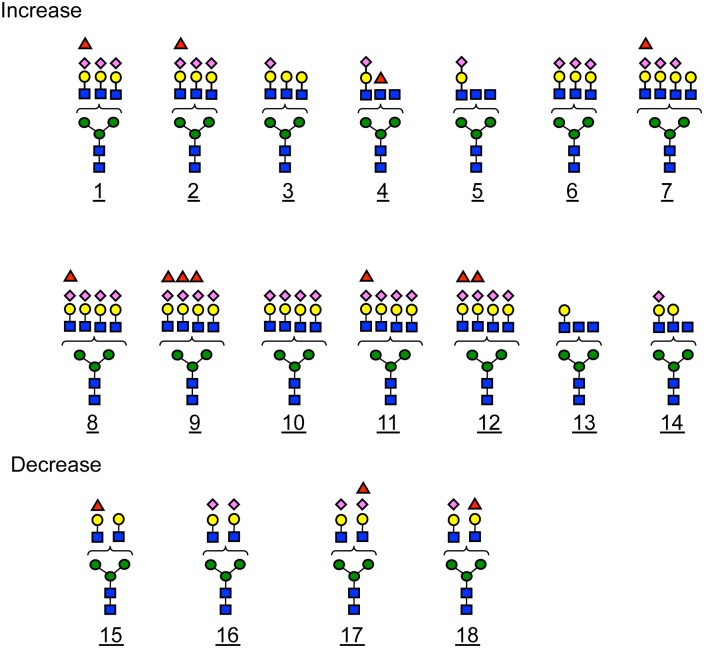
Deduced structures of characteristic *N*-glycans in SSCs. The number of the *N*-glycan corresponded to Table 1. No. 1~14 were increased and No. 15~18 were decreased in SSCs, respectively. blue square, *N*-acetylglucosamine; yellow circle, galactose; green circle, mannose; purple diamond, *N*-acetylneuraminic acid; red triangle, fucose.

Data obtained from the positive and negative ion modes were combined, and O-PLS was performed using SIMCA P+ 12.0.1. The score plot (Fig 2B) and loading plot (Fig 2C) obtained by O-PLS between young controls, aged controls, and SSCs were shown. As shown in the score plots (Fig 2B), the groups of young controls (shadowed symbol), aged controls (open symbols), and SSCs (closed symbols) were more clearly separated than in PCA (R2X [1] = 0.295051, R2X [XSide Comp. 1] = 0.106066, Ellipse: Hotelling (T2) 0.95). Furthermore, Y-variation (age) and Y-prediction (age) strongly correlated (R2 = 0.9556) in the scatter plot (S3 Fig), suggesting that the O-PLS model was appropriate for the prediction of each age on the basis of *N*-glycans. Therefore, the characteristic *N*-glycan structures in SSCs may be successfully discriminated using this model. Thus, the characteristic *N*-glycan structures in SSCs, which contributed to the classification of SSCs from young/aged controls, may be selected from the loading plot data (Fig 2C). The absolute value of [pq1], on the vertical axis of Fig 2C, represents the weight that combined the X variable (the peak area ratio of each *N*-glycan to all identified *N*-glycans) with the Y-variable (age). The horizontal axis of Fig 2C shows the glycan compositions deduced from the observed *m/z* of each *N*-glycan. The ratio of the absolute [pq1] value to the corresponding error was summarized in S2 Table. *N*-glycans that carrying this value larger than 1.5 were considered to be characteristic *N*-glycans in SSCs (closed columns shown in Fig 2C). Since dHex1Hex6HexNAc5NeuNAc3 (1), dHex1Hex6HexNAc5NeuNAc3 (2), Hex6HexNAc5NeuNAc3 (1), Hex5HexNAc4NeuNAc2 (1), and dHex1Hex5HexNAc4NeuNAc2 were detected in both ion modes, the higher absolute pq[1] value was chosen. The positive and negative values of [pq1] indicated increased and decreased *N*-glycans in SSCs, respectively. The increased and decreased *N*-glycans in SSCs thus obtained were listed in order of increasing absolute pq[1] values (Table 1), and their deduced structures were shown in Fig 3. The MS/MS data of each *N*-glycan were shown in S4 Fig. Taken together, the results obtained demonstrated that multi-branched and highly sialylated *N*-glycans (No. 1, 2, and 6–12 in Fig 3) and agalacto- and/or bisecting- *N*-glycans (No. 4, 5, 13, and 14 in Fig 3) were increased in SSCs. On the other hand, biantennary *N*-glycans (No. 15–18 in Fig 3) were decreased in SSCs.

**Table 1:** Summary of characteristic *N*-glycan profiles in SSCs.

No.^a^	Observed *m/z* value^b^	Charge state	Observed exact mass	Calculated exact mass	Glycan composition^c^
1	1514.57(1)	2+	3027.14	3027.08	dHex1 Hex6 HexNAc5 NeuNAc3
2	1514.57(2)	2+	3027.14	3027.08	dHex1 Hex6 HexNAc5 NeuNAc3
	1512.55(2)	2-	3027.10	3027.08	dHex1 Hex6 HexNAc5 NeuNAc3
3	1150.44(2)	2+	2298.88	2298.84	Hex6 HexNAc5 NeuNAc1
4	1061.41	2+	2120.82	2120.79	dHex1 Hex4 HexNAc5 NeuNAc1
5	988.37	2+	1974.74	1974.73	Hex4 HexNAc5 NeuNAc1
6	1439.52 (1)	2-	2881.04	2881.03	Hex6 HexNAc5 NeuNAc3
7	1695.12	2-	3392.24	3392.22	dHex1 Hex7 HexNAc6 NeuNAc3
8	1226.77(2)	3-	3683.31	3683.31	dHex1 Hex7 HexNAc6 NeuNAc4
9	1324.14	3-	3975.42	3975.43	dHex3 Hex7 HexNAc6 NeuNAc4
10	1178.09(2)	3-	3537.27	3537.25	Hex7 HexNAc6 NeuNAc4
11	1226.77(1)	3-	3683.31	3683.31	dHex1 Hex7 HexNAc6 NeuNAc4
12	1275.47(2)	3-	3829.41	3829.37	dHex2 Hex7 HexNAc6 NeuNAc4
13	842.82	2+	1683.64	1683.63	Hex4 HexNAc5
14	1069.4	2+	2136.80	2136.78	Hex5 HexNAc5 NeuNAc1
15	895.34	2+	1788.68	1788.67	dHex1 Hex5 HexNAc4
16	1111.39(1)	2-	2224.78	2224.80	Hex5 HexNAc4 NeuNAc2
17	1184.43	2-	2370.86	2370.86	dHex1 Hex5 HexNAc4 NeuNAc2
18	1040.89	2+	2079.78	2079.76	dHex1 Hex5 HexNAc4 NeuNAc1
	1038.88	2-	2079.76	2079.76	dHex1 Hex5 HexNAc4 NeuNAc1

Increased and decreased *N-*glycans in SSCs were sorted by pq[1] values in the loading plot of O-PLS shown in Fig 2C.

^a^ The number of the *N*-glycan corresponded to Figs 3 and S7.

^b^ The number in parentheses indicates isomers.

^c^ Hex, hexose; HexNAc, *N*-acetylhexosamine; NeuNAc, *N*-acetylneuraminic acid; dHex, deoxyhexose.

While we analyzed combined data obtained from both ion modes, the peak area ratios may not have been compatible with each ion mode. Therefore, we performed O-PLS separately using the data obtained from each ion mode. As shown in S5 Fig (positive ion mode) and S6 Fig (negative ion mode), the characteristic *N*-glycans in SSCs were consistent with the results obtained using the combined data of both ion modes (Table 1). Therefore, the combined O-PLS analyses of data obtained from the positive and negative ion modes were considered to be very useful and reliable.

### Confirmation of Characteristic *N*-Glycans in SSCs Using Analyses of Variance (ANOVA)

We performed a one-way analyses of variance (ANOVA) on the peak area ratios of each *N*-glycan, which were detected via MVA. The results obtained indicated that in all *N*-glycans, except for No. 13 and 14, the means of the peak area ratios were significantly different among the three groups tested. Since *N*-glycans of No. 13 and 14 showed the lowest pq[1] values by O-PLS (Fig 2C and S2 Table), both may be the least important of the characteristic *N*-glycans in SSCs. Multiple comparisons to SSCs were performed using Dunnett’s test, as shown in S7A and S7B Fig. Although no significant difference (*p* < 0.05) was observed in some *N*-glycans between SSCs and aged controls, slight increases or decreases was detected in SSCs.

### Contents of Sialic Acid in Plasma *N*-Glycans

The contents of sialic acid in the plasma *N*-glycans of SSCs and aged controls were determined using a sialic acid (NANA) fluorometric assay. After the preparation of *N*-glycans from plasma proteins, as described in Materials and Methods section, sialic acid was released by an incubation with *A*. *ureafaciens* sialidase. Sialic acid in plasma *N*-glycans was significantly higher in SSCs than in aged controls (SSC; 5.16 ± 0.43, aged; 3.51 ± 0.24* nmol/mg protein. Data are represented as the mean ± SE (n = 8). *p < 0.05, different from SSC). These results were consistent with the increase in sialylated *N*-glycans in SSCs obtained by LC/MS.

### Plasma Biomarkers

Plasma biomarkers in SSCs and the controls were measured (Table 2). Albumin levels, the most documented predictor of health outcomes in older individuals, were significantly lower in SSCs than in young and aged controls. On the other hand, the levels of inflammatory agents, such as CRP, adiponectin, IL-6, and TNF-α, were all elevated in SSCs.

**Table 2:** Comparison of physiological and inflammatory parameters between SSCs and young/aged controls.

	Young (5)^c^	Aged (5)	SSCs (6)
Age^a^ (years)	30.2 ± 8.11	71.6 ± 1.52	106.7 ± 0.52
Albumin^a^ (g/dL)	4.82 ± 0.31**^d^	4.28 ± 0.08*^d^	3.82 ± 0.31
CRP^b^ (mg/dL)	0.03 (0.02–0.03)*^e^	0.03 (0.03–0.04)*^e^	0.22 (0.06–0.31)
Adiponectin^b^ (μg/mL)	ND^f^	15.96 (11.11–22.91)	27.35 (16.00–36.30)
IL-6^b^ (pg/mL)	ND ^f^	1.22 (0.97–1.49)*^e^	3.90 (2.38–5.42)
TNF-α^b^ (pg/mL)	ND ^f^	2.11 (2.00–2.23)**^e^	3.74 (3.61–4.75)

^a^Data represent the mean± standard deviation.

^**b**^Data represent the median (interquartile range).

^**c**^Numbers in parentheses indicate the numbers of subjects.

^d^Differences from SSCs were calculated by ANOVA (*p < 0.05, **p < 0.01).

^e^Differences from SSCs were calculated by ANOVA using logarithmically transformed data (*p < 0.05, **p < 0.01).

^f^not determined

## Discussion

In the present study, we investigated longevity-associated *N*-glycans in SSCs. We performed an *N*-glycomic analysis of plasma proteins in SSCs, a model of human longevity, using LC/MS and O-PLS. The results obtained showed that multi-branched and highly sialylated *N*-glycans as well as agalacto- and/or bisecting *N*-glycans were higher, whereas biantennary *N*-glycans were lower in SSCs than in young and aged controls. This is the first study to demonstrate that *N*-glycans are associated with extreme longevity and healthy aging in humans.

The effects of aging on *N*-glycan profiles in human plasma or serum were recently examined using several methods: DNA sequencer-assisted, fluorophore-assisted carbohydrate electrophoresis (DSA-FACE) [16–20], nano-HPLC-chip-TOF-MS [21, 22], UPLC and MALDI-TOF-MS [23], and hydrophilic interaction high performance liquid chromatography (HILIC) [6, 24–26]. All studies found age-dependent increases in non-galactosylated biantennary *N*-glycans and corresponding decreases in digalactosylated biantennary *N*-glycans [16, 20, 24, 25]. Additionally, specific glycoproteins, such as IgG, instead of total plasma glycoproteins were reported to show similar alterations with aging [27, 28]. In the present study on SSCs, a reduction was also observed in digalactosyl biantennary *N*-glycan (No. 15 in Fig 3), suggesting that decrease in the *N*-glycan is age-associated changes.

On the other hand, the increases in multi-branched and highly sialylated *N*-glycans (No. 1, 2, 6–12 in Fig 3) observed in SSCs were not reported in previous studies on aging. Therefore, these changes may be associated with extreme longevity. Previous studies demonstrated that multi-branched and highly sialylated *N*-glycans were elevated in response to inflammatory diseases, such as rheumatoid arthritis [29], ulcerative colitis [30], and chronic pancreatitis [31]. However, no subjects in the present study had or had previous histories of inflammatory diseases. On the other hand, chronic inflammation is known to be enhanced with healthy aging [32], and we found that inflammatory factors such as CRP, adiponectin, TNF-α, and IL-6 were elevated in SSCs (Table 2). Therefore, multi-branched and highly sialylated *N*-glycans may be increased in SSCs as a response to chronic inflammation. The structures of sialic acids, sialyl linkages (α2,3 or α2,6), and *O*-acetylated forms [33–35] need to be considered because their differences may be relevant to the biological role of plasma glycoproteins in inflammation.

In the Leiden Longevity study, Ruhaak et al. examined longevity-associated *N*-glycans and reported that non-fucosylated biantennary glycans were significantly higher in the offspring of nonagenarians (90 years old) than in controls [25]. However, our results obtained from SSCs were not consistent with these findings. A difference may exist between nonagenarians and SSCs. Nevertheless, a follow-up of individual offspring is awaited in order to determine whether they will also have long lifespans. This information is needed in order to establish whether the *N*-glycans described above are associated with longevity.

No significant feature of fucosylated glycans was observed in the present study. The fucosylation of *N*-glycans has been implicated in several diseases including cancer [36]. Since the subjects who participated in this study were healthy individuals, fucosylated *N*-glycans in SSCs may not have been altered.

Human longevity may be associated with genes [1]. Several studies have attempted to discover the genetic basis of extreme longevity [37, 38]. A genome-wide association study (GWAS) of *N*-glycomes in human plasma revealed polymorphisms in several glycogenes [39]; one was *MGAT5*, which codes for the enzyme involved in the generation of multi-branched *N*-glycans, while another was *SLC9A9*, which codes for a proton pump affecting Golgi pH linked to the sialylation of glycans. Further studies are warranted in order to determine whether these genes show any polymorphisms in SSCs, the findings of which may contribute to a better understanding of the roles of glycans in human longevity.

In conclusion, we herein identified an enhancement in multi-branched and highly sialylated *N*-glycans in SSCs. These *N*-glycan changes may play a role in anti-inflammatory responses against enhanced chronic inflammation. Thus, proper responses to inflammation through *N*-glycan modulations may be key for longevity and healthy aging in human. Future studies, including those involving different genders and ethnicities, are needed in order to obtain more detailed insights into the biological resilience of SSCs.

## Supporting Information

S1 Fig Base peak chromatograms of reduced *N*-glycans from human plasma. Base peak chromatograms of 5 young controls, 5 aged controls, and 6 SSCs were obtained from LC/MS^n^. Vertical and horizontal axes of each panel represent the relative abundance and retention time of LC/MS, respectively. (A) positive ion mode, (B) negative ion mode.
(PDF)

S2 Fig Peak area ratio of each *N*-glycan. The peak area ratio of each *N*-glycan was calculated against the total peak area of all identified *N*-glycans in the (A) positive and (B) negative ion modes. Shadowed, open, and closed columns represent young controls, aged controls, and SSCs, respectively. "Glycan composition" represents each deduced glycan composition from mass spectra. Numbers in parentheses indicate isomers. Data represent the mean ± standard deviation (SD) (n = 5 in young and aged, n = 6 in SSC). Row data were summarized in S1 Table. Hex, hexose; HexNAc, *N*-acetylhexosamine; NeuNAc, *N*-acetylneuraminic acid; dHex, deoxyhexose; NH_4_, ammonium.
(PDF)

S3 Fig Correlation between the Y Variation (Age) and Y Prediction (Age) obtained by O-PLS. Regression equation; y = 1*x-3.393e-006; R2 = 0.9556. Shadowed symbols, young; open symbols, aged; closed symbols, SSCs.
(PDF)

S4 Fig Fragmentation of MS/MS spectra from *N*-glycans. Spectra represent increased and decreased *N*-glycans in SSCs. *N*-glycans not shown here were deduced by each calculated exact mass. Numbers of the *N*-glycans correspond to Table 1. blue square, *N*-acetylglucosamine; yellow circle, galactose; green circle, mannose; purple diamond, *N*-acetylneuraminic acid; red triangle, fucose.
(PDF)

S5 Fig Score plot and loading plot of O-PLS using data from the positive ion mode. (A) An O-PLS score plot between young controls (shadowed), aged controls (opened), and SSCs (closed). R2X [1] = 0.263562, R2X [XSide Comp. 1] = 0.205859, Ellipse: Hotelling T2 (95%). (B) A loading plot by O-PLS between young controls, aged controls, and SSCs. Numbers in parentheses indicate isomers. "P" in parentheses indicates data obtained from the positive ion mode. Closed and shadowed columns represent [pq1] / SE > 1.5 and < 1.5, respectively. Hex, hexose; HexNAc, *N*-acetylhexosamine; NeuNAc, *N*-acetylneuraminic acid; dHex, deoxyhexose; NH_4_, ammonium.
(PDF)

S6 Fig Score plot and loading plot of O-PLS using data from the negative ion mode. (A) An O-PLS score plot between young controls (shadowed), aged controls (opened), and SSCs (closed). R2X [1] = 0.416393, R2X [XSide Comp. 1] = 0.241192, Ellipse: Hotelling T2 (95%). (B) A loading plot by O-PLS between young controls, aged controls, and SSCs. Numbers in parentheses indicate isomers. "N" in parentheses indicates data obtained from the negative ion mode. Closed and shadowed columns represent [pq1] / SE > 1.5 and < 1.5, respectively. Hex, hexose; HexNAc, *N*-acetylhexosamine; NeuNAc, *N*-acetylneuraminic acid; dHex, deoxyhexose.
(PDF)

S7 Fig Age alterations in peak area ratios of each *N*-glycan summarized in Table 1. The numbers of graphs correspond to the glycan numbers in Table 1. The vertical axis indicates peak area ratios against the total peak area of all *N*-glycans in each ion mode. In the case of No. 2 and No.18, the data of the negative ion mode were used because the absolute pq[1] values in the negative mode were larger than that in the positive mode (shown in Fig 2C). Row data were summarized in S2 Table. Data represent the mean ± SD (n = 5 in young and aged, n = 6 in SSC). (A) Increased *N*-glycans in SSCs, (B) Decreased *N*-glycans in SSCs, *p < 0.05, **p < 0.01, vs SSC.
(PDF)

S1 Table Retention time and peak area ratio of each *N*-glycan detected in positive and negative ion modes. ^1^Numbers in parentheses represent isomers, and "N" or "P" in parentheses indicates the data obtained from negative or positive ion mode, respectively. ^2^Hex, hexose; HexNAc, *N*-acetylhexosamine; NeuNAc, *N*-acetylneuraminic acid; dHex, deoxyhexose; NH4, ammonium. ^3^RT represents retention time.
(PDF)

S2 Table Weight (pq[1]) and its error (SE) of each *N*-glycan calculated using O-PLS. ^1^Numbers in parentheses represent isomers, and "N" or "P" in parentheses indicates data obtained from the negative or positive ion mode, respectively. ^2^Hex, hexose; HexNAc, *N*-acetylhexosamine; NeuNAc, *N*-acetylneuraminic acid; dHex, deoxyhexose; NH4, ammonium.
(PDF)

## Abbreviations

SSC: semisupercentenarian
O-PLS: orthogonal projections to latent structures
PCA: principle component analyses
LC/MS^n^: Liquid chromatography/multiple stage mass spectrometry
MVA: multivariate analysis
CRP: C-reactive protein
IL-6: interleukin-6
TNF-α: tumor necrosis factor-α
Con A: concanavalin A

## References

[1] Lopez-Otin C, Blasco MA, Partridge L, Serrano M, Kroemer G. The hallmarks of aging. Cell. 2013;153(6):1194–217. 10.1016/j.cell.2013.05.039 23746838 PMC3836174

[2] Arai Y, Inagaki H, Takayama M, Abe Y, Saito Y, Takebayashi T, et al Physical independence and mortality at the extreme limit of life span: supercentenarians study in Japan. The journals of gerontology Series A, Biological sciences and medical sciences. 2014;69(4):486–94. 10.1093/gerona/glt146 . 24225329

[3] Miura Y, Sato Y, Arai Y, Abe Y, Takayama M, Toda T, et al Proteomic analysis of plasma proteins in Japanese semisuper centenarians. Experimental gerontology. 2011;46(1):81–5. 10.1016/j.exger.2010.10.002 . 20951194

[4] Callewaert N, Schollen E, Vanhecke A, Jaeken J, Matthijs G, Contreras R. Increased fucosylation and reduced branching of serum glycoprotein N-glycans in all known subtypes of congenital disorder of glycosylation I. Glycobiology. 2003;13(5):367–75. 10.1093/glycob/cwg040 . 12626389

[5] Freeze HH, Aebi M. Altered glycan structures: the molecular basis of congenital disorders of glycosylation. Current opinion in structural biology. 2005;15(5):490–8. 10.1016/j.sbi.2005.08.010 . 16154350

[6] Lu JP, Knezevic A, Wang YX, Rudan I, Campbell H, Zou ZK, et al Screening novel biomarkers for metabolic syndrome by profiling human plasma N-glycans in Chinese Han and Croatian populations. Journal of proteome research. 2011;10(11):4959–69. 10.1021/pr2004067 . 21939225

[7] Ruhaak LR, Koeleman CA, Uh HW, Stam JC, van Heemst D, Maier AB, et al Targeted biomarker discovery by high throughput glycosylation profiling of human plasma alpha1-antitrypsin and immunoglobulin A. PloS one. 2013;8(9):e73082 10.1371/journal.pone.0073082 24039863 PMC3767703

[8] Sato Y, Endo T. Alteration of brain glycoproteins during aging. Geriatrics & gerontology international. 2010;10 Suppl 1:S32–40. 10.1111/j.1447-0594.2010.00602.x . 20590840

[9] Hashii N, Kawasaki N, Itoh S, Hyuga M, Kawanishi T, Hayakawa T. Glycomic/glycoproteomic analysis by liquid chromatography/mass spectrometry: analysis of glycan structural alteration in cells. Proteomics. 2005;5(18):4665–72. 10.1002/pmic.200401330 . 16281179

[10] Itoh S, Kawasaki N, Hashii N, Harazono A, Matsuishi Y, Hayakawa T, et al N-linked oligosaccharide analysis of rat brain Thy-1 by liquid chromatography with graphitized carbon column/ion trap-Fourier transform ion cyclotron resonance mass spectrometry in positive and negative ion modes. Journal of chromatography A. 2006;1103(2):296–306. 10.1016/j.chroma.2005.11.043 . 16364349

[11] Hashii N, Harazono A, Kuribayashi R, Takakura D, Kawasaki N. Characterization of N-glycan heterogeneities of erythropoietin products by liquid chromatography/mass spectrometry and multivariate analysis. Rapid communications in mass spectrometry: RCM. 2014;28(8):921–32. 10.1002/rcm.6858 . 24623697

[12] Kaneshiro N, Xiang Y, Nagai K, Kurokawa MS, Okamoto K, Arito M, et al Comprehensive analysis of short peptides in sera from patients with IgA nephropathy. Rapid communications in mass spectrometry: RCM. 2009;23(23):3720–8. 10.1002/rcm.4315 . 19902551

[13] Tsutsui H, Maeda T, Min JZ, Inagaki S, Higashi T, Kagawa Y, et al Biomarker discovery in biological specimens (plasma, hair, liver and kidney) of diabetic mice based upon metabolite profiling using ultra-performance liquid chromatography with electrospray ionization time-of-flight mass spectrometry. Clinica chimica acta; international journal of clinical chemistry. 2011;412(11–12):861–72. 10.1016/j.cca.2010.12.023 . 21185819

[14] Holm T, Rutishauser D, Kai-Larsen Y, Lyutvinskiy Y, Stenius F, Zubarev RA, et al Protein biomarkers in vernix with potential to predict the development of atopic eczema in early childhood. Allergy. 2014;69(1):104–12. 10.1111/all.12308 24205894 PMC4226386

[15] Dutta M, Subramani E, Taunk K, Gajbhiye A, Seal S, Pendharkar N, et al Investigation of serum proteome alterations in human endometriosis. Journal of proteomics. 2015;114:182–96. 10.1016/j.jprot.2014.10.021 . 25449831

[16] Vanhooren V, Desmyter L, Liu XE, Cardelli M, Franceschi C, Federico A, et al N-glycomic changes in serum proteins during human aging. Rejuvenation research. 2007;10(4):521–31a. 10.1089/rej.2007.0556 . 18047421

[17] Vanhooren V, Laroy W, Libert C, Chen C. N-glycan profiling in the study of human aging. Biogerontology. 2008;9(5):351–6. 10.1007/s10522-008-9140-z . 18431686

[18] Vanhooren V, Liu XE, Franceschi C, Gao CF, Libert C, Contreras R, et al N-glycan profiles as tools in diagnosis of hepatocellular carcinoma and prediction of healthy human ageing. Mechanisms of ageing and development. 2009;130(1–2):92–7. 10.1016/j.mad.2008.11.008 . 19070631

[19] Vanhooren V, Dewaele S, Libert C, Engelborghs S, De Deyn PP, Toussaint O, et al Serum N-glycan profile shift during human ageing. Experimental gerontology. 2010;45(10):738–43. 10.1016/j.exger.2010.08.009 . 20801208

[20] Ding N, Nie H, Sun X, Sun W, Qu Y, Liu X, et al Human serum N-glycan profiles are age and sex dependent. Age and ageing. 2011;40(5):568–75. 10.1093/ageing/afr084 . 21807702

[21] Ruhaak LR, Miyamoto S, Kelly K, Lebrilla CB. N-Glycan profiling of dried blood spots. Analytical chemistry. 2012;84(1):396–402. 10.1021/ac202775t 22128873 PMC3259271

[22] Aldredge D, An HJ, Tang N, Waddell K, Lebrilla CB. Annotation of a serum N-glycan library for rapid identification of structures. Journal of proteome research. 2012;11(3):1958–68. 10.1021/pr2011439 22320385 PMC3292799

[23] Lauc G, Huffman JE, Pucic M, Zgaga L, Adamczyk B, Muzinic A, et al Loci associated with N-glycosylation of human immunoglobulin G show pleiotropy with autoimmune diseases and haematological cancers. PLoS Genet. 2013;9(1):e1003225 10.1371/journal.pgen.1003225 23382691 PMC3561084

[24] Knezevic A, Gornik O, Polasek O, Pucic M, Redzic I, Novokmet M, et al Effects of aging, body mass index, plasma lipid profiles, and smoking on human plasma N-glycans. Glycobiology. 2010;20(8):959–69. 10.1093/glycob/cwq051 . 20356825

[25] Ruhaak LR, Uh HW, Beekman M, Hokke CH, Westendorp RG, Houwing-Duistermaat J, et al Plasma protein N-glycan profiles are associated with calendar age, familial longevity and health. Journal of proteome research. 2011;10(4):1667–74. 10.1021/pr1009959 . 21184610

[26] Saldova R, Asadi Shehni A, Haakensen VD, Steinfeld I, Hilliard M, Kifer I, et al Association of N-glycosylation with breast carcinoma and systemic features using high-resolution quantitative UPLC. Journal of proteome research. 2014;13(5):2314–27. 10.1021/pr401092y . 24669823

[27] Parekh R, Roitt I, Isenberg D, Dwek R, Rademacher T. Age-related galactosylation of the N-linked oligosaccharides of human serum IgG. J Exp Med. 1988;167(5):1731–6. 3367097 10.1084/jem.167.5.1731 PMC2188930

[28] Tsuchiya N, Endo T, Matsuta K, Yoshinoya S, Takeuchi F, Nagano Y, et al Detection of glycosylation abnormality in rheumatoid IgG using N-acetylglucosamine-specific Psathyrella velutina lectin. Journal of immunology. 1993;151(2):1137–46. . 8335895

[29] Pawlowski T, Mackiewicz A, Mackiewicz S. Studies on microheterogeneity of acute-phase proteins in rheumatoid arthritis by using crossed affinoimmuno-electrophoresis with free concanavalin A. Behring Institute Mitteilungen. 1986;(80):11–5. . 2428350

[30] Miyahara K, Nouso K, Saito S, Hiraoka S, Harada K, Takahashi S, et al Serum glycan markers for evaluation of disease activity and prediction of clinical course in patients with ulcerative colitis. PloS one. 2013;8(10):e74861 10.1371/journal.pone.0074861 24116015 PMC3792068

[31] Sarrats A, Saldova R, Pla E, Fort E, Harvey DJ, Struwe WB, et al Glycosylation of liver acute-phase proteins in pancreatic cancer and chronic pancreatitis. Proteomics Clinical applications. 2010;4(4):432–48. 10.1002/prca.200900150 . 21137062

[32] Franceschi C, Bonafe M, Valensin S, Olivieri F, De Luca M, Ottaviani E, et al Inflamm-aging. An evolutionary perspective on immunosenescence. Ann N Y Acad Sci. 2000;908:244–54. . 10911963 10.1111/j.1749-6632.2000.tb06651.x

[33] Gagneux P, Cheriyan M, Hurtado-Ziola N, van der Linden EC, Anderson D, McClure H, et al Human-specific regulation of alpha 2-6-linked sialic acids. The Journal of biological chemistry. 2003;278(48):48245–50. 10.1074/jbc.M309813200 . 14500706

[34] Hedlund M, Ng E, Varki A, Varki NM. alpha 2-6-Linked sialic acids on N-glycans modulate carcinoma differentiation in vivo. Cancer Res. 2008;68(2):388–94. 10.1158/0008-5472.CAN-07-1340 . 18199532

[35] Langereis MA, Bakkers MJ, Deng L, Padler-Karavani V, Vervoort SJ, Hulswit RJ, et al Complexity and Diversity of the Mammalian Sialome Revealed by Nidovirus Virolectins. Cell Rep. 2015;11(12):1966–78. 10.1016/j.celrep.2015.05.044 . 26095364 PMC5292239

[36] Taniguchi N, Kizuka Y. Glycans and cancer: role of N-glycans in cancer biomarker, progression and metastasis, and therapeutics. Adv Cancer Res. 2015;126:11–51. 10.1016/bs.acr.2014.11.001 . 25727145

[37] Sebastiani P, Solovieff N, Dewan AT, Walsh KM, Puca A, Hartley SW, et al Genetic signatures of exceptional longevity in humans. PloS one. 2012;7(1):e29848 10.1371/journal.pone.0029848 22279548 PMC3261167

[38] Gierman HJ, Fortney K, Roach JC, Coles NS, Li H, Glusman G, et al Whole-genome sequencing of the world's oldest people. PloS one. 2014;9(11):e112430 10.1371/journal.pone.0112430 25390934 PMC4229186

[39] Huffman JE, Knezevic A, Vitart V, Kattla J, Adamczyk B, Novokmet M, et al Polymorphisms in B3GAT1, SLC9A9 and MGAT5 are associated with variation within the human plasma N-glycome of 3533 European adults. Human molecular genetics. 2011;20(24):5000–11. 10.1093/hmg/ddr414 . 21908519

