# Supplementary figures and images for: Change in N-Glycosylation of Plasma Proteins in Japanese Semisupercentenarians
Source: PLoS One. 2015 Nov 11;10(11):e0142645. doi: 10.1371/journal.pone.0142645 (PMC4641608; doi:10.1371/journal.pone.0142645)

S1 Fig.  
(A)

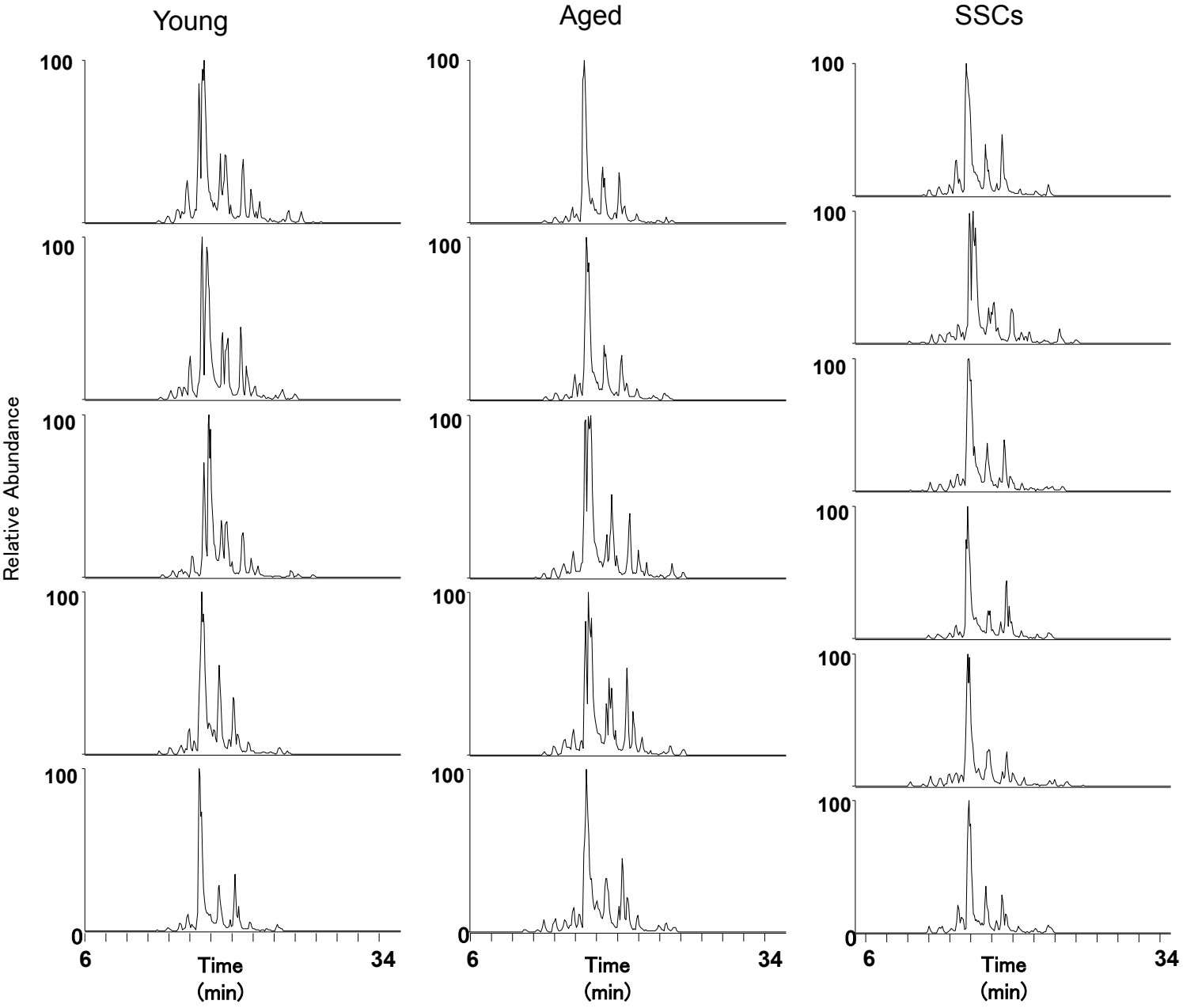

(B)

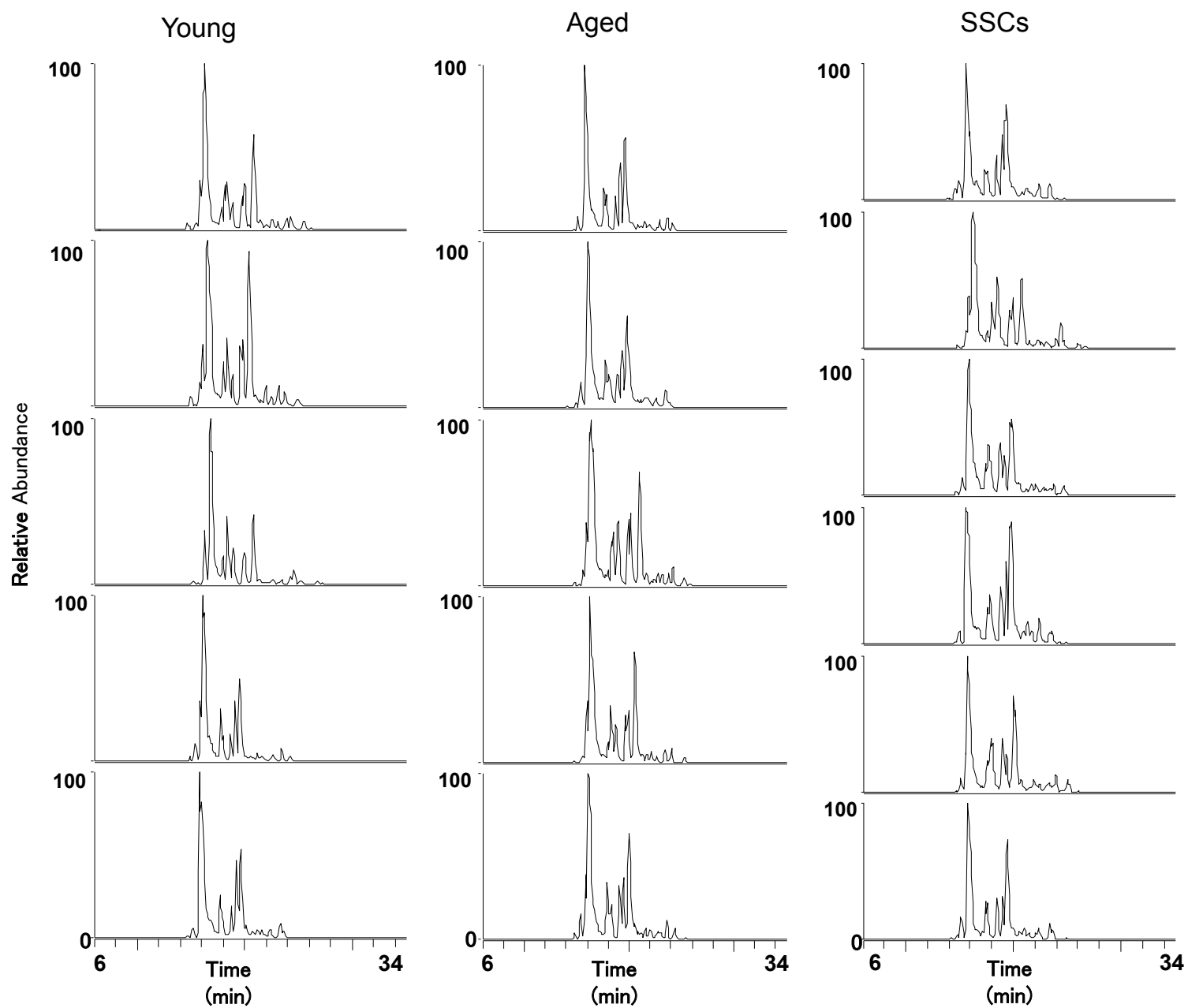

Supplement: S1 Fig — Base peak chromatograms of 5 young controls, 5 aged controls, and 6 SSCs were obtained from LC/MSn. Vertical and horizontal axes of each panel represent the relative abundance and retention time of LC/MS, respectively. (A) positive ion mode, (B) negative ion mode. (PDF) [file pone.0142645.s001.pdf]

S2 Fig.

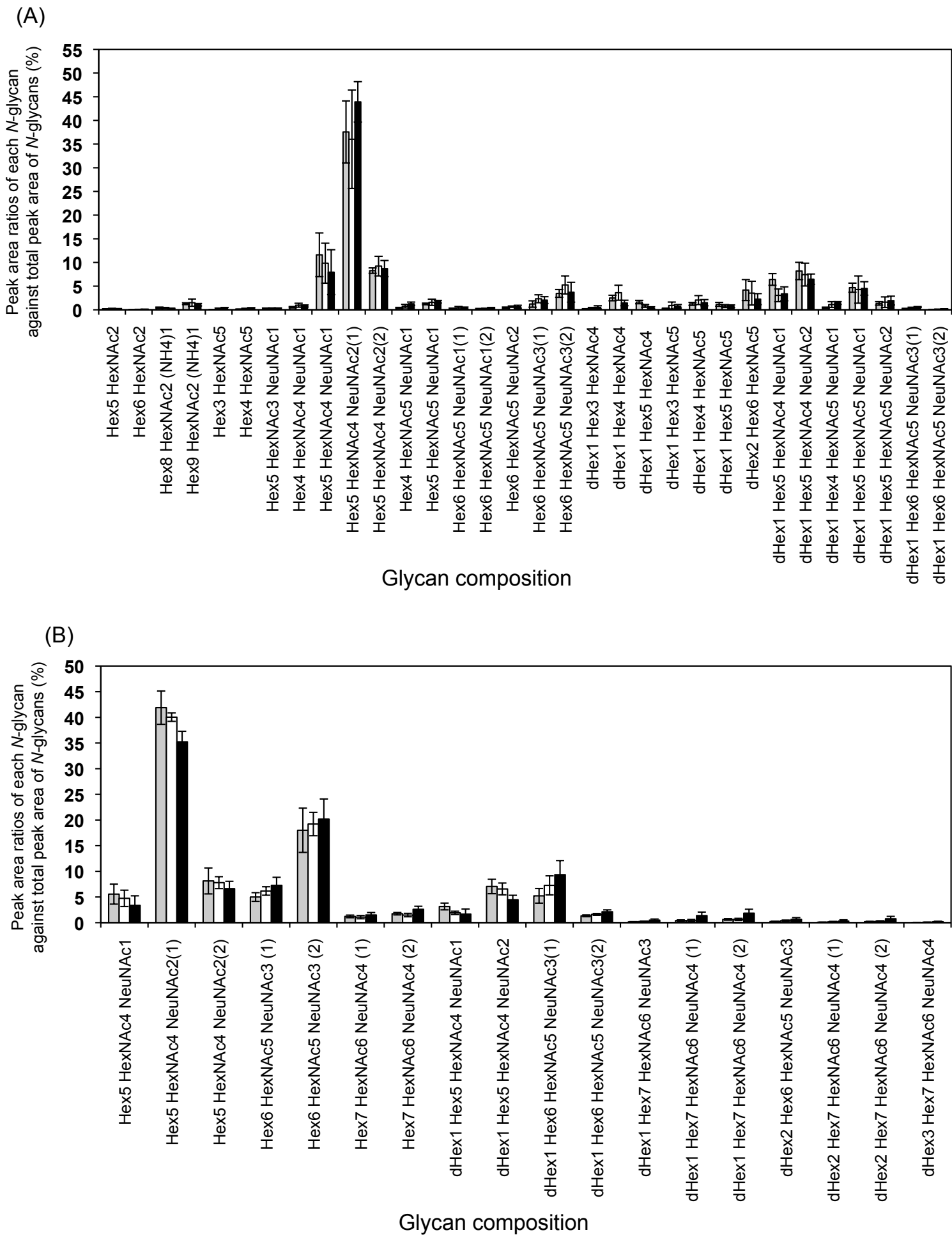

Supplement: S2 Fig — The peak area ratio of each N-glycan was calculated against the total peak area of all identified N-glycans in the (A) positive and (B) negative ion modes. Shadowed, open, and closed columns represent young controls, aged controls, and SSCs, respectively. "Glycan composition" represents each deduced glycan composition from mass spectra. Numbers in parentheses indicate isomers. Data represent the mean ± standard deviation (SD) (n = 5 in young and aged, n = 6 in SSC). Row data were summarized in S1 Table. Hex, hexose; HexNAc, N-acetylhexosamine; NeuNAc, N-acetylneuraminic acid; dHex, deoxyhexose; NH4, ammonium. (PDF) [file pone.0142645.s002.pdf]

S3 Fig.

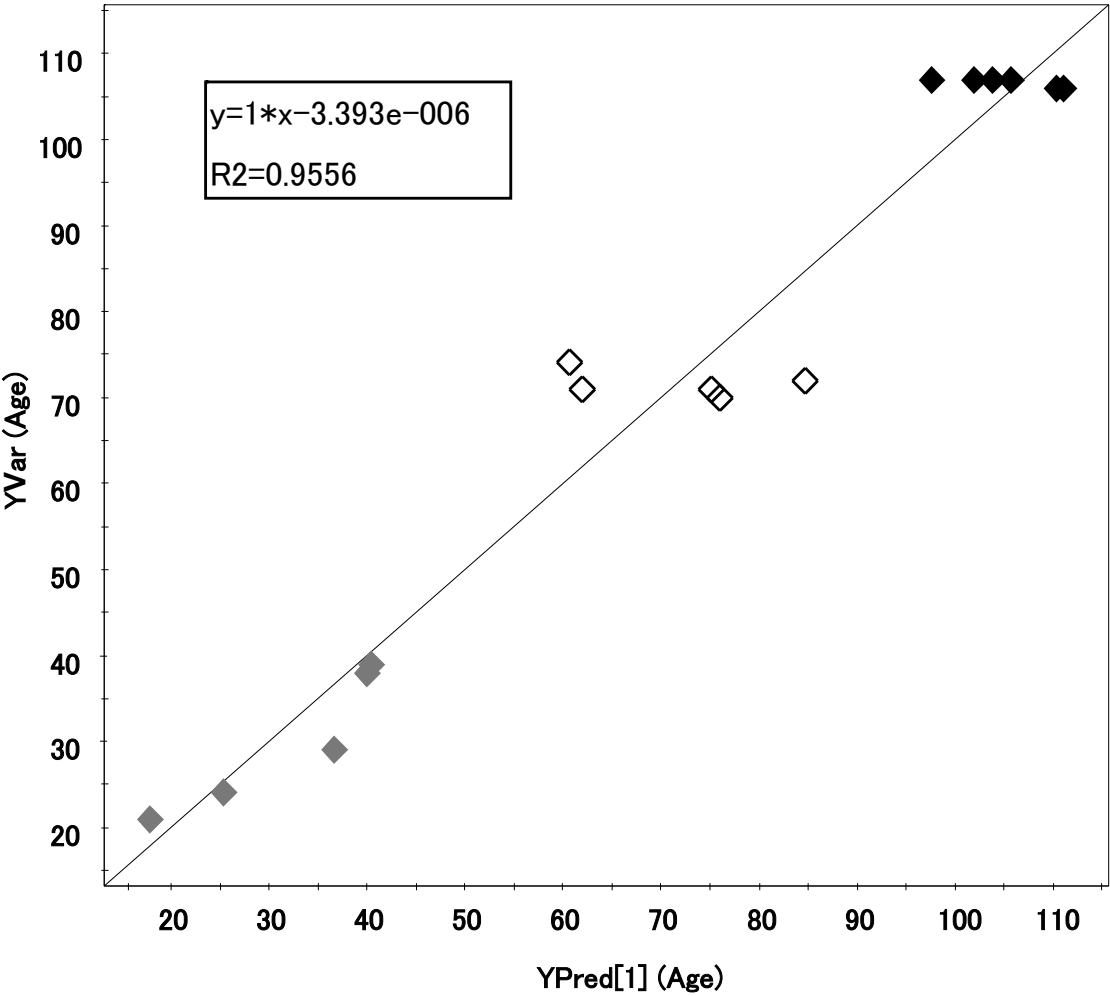

Supplement: S3 Fig — Regression equation; y = 1*x-3.393e-006; R2 = 0.9556. Shadowed symbols, young; open symbols, aged; closed symbols, SSCs. (PDF) [file pone.0142645.s003.pdf]

**Fig.S4** Increased glycans

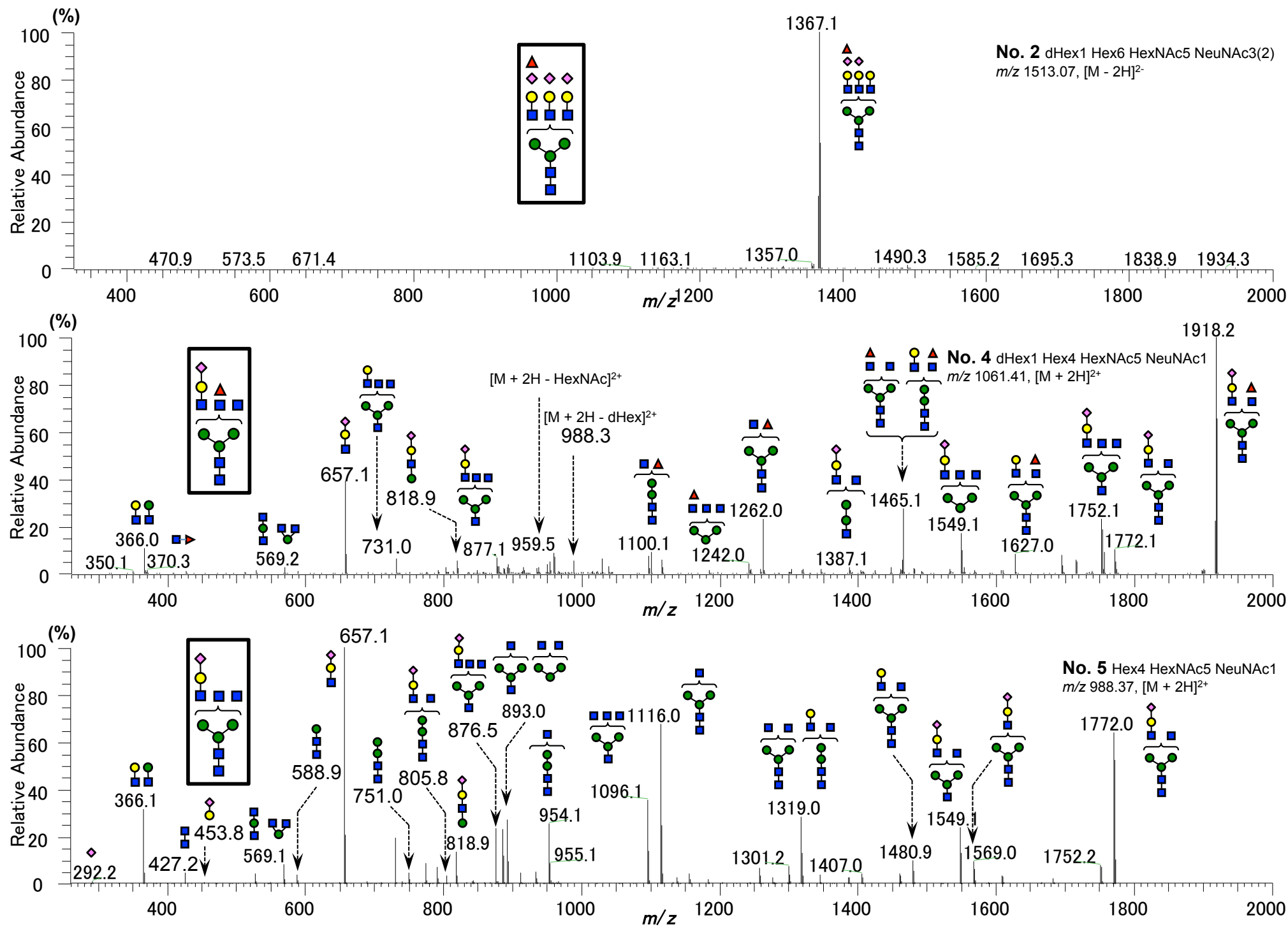

# Increased glycans

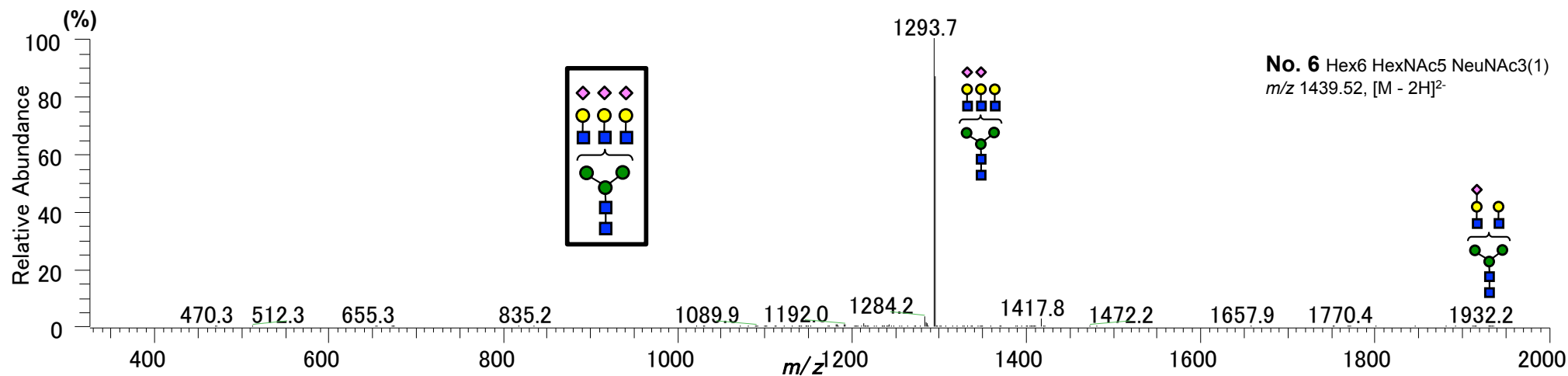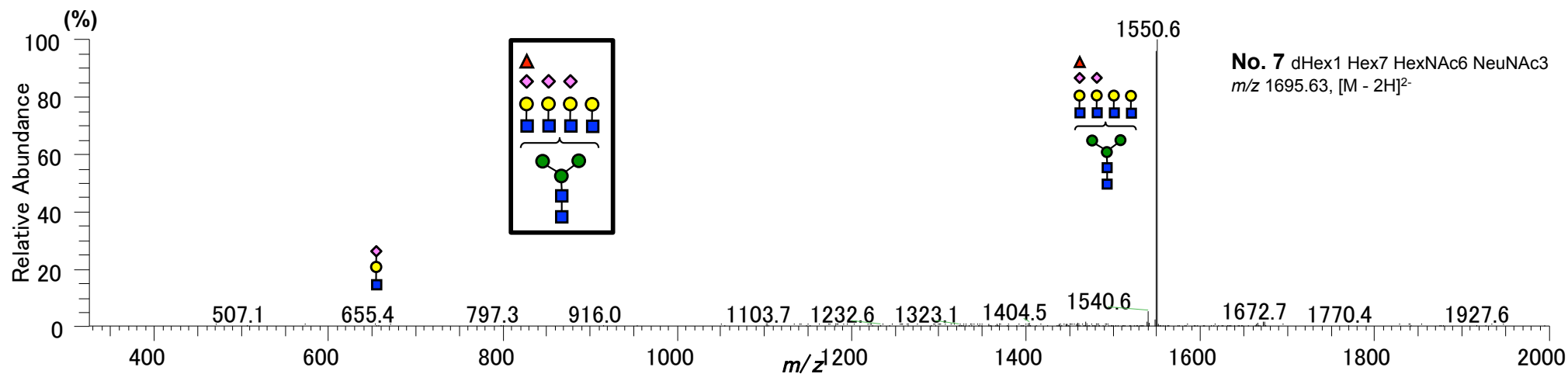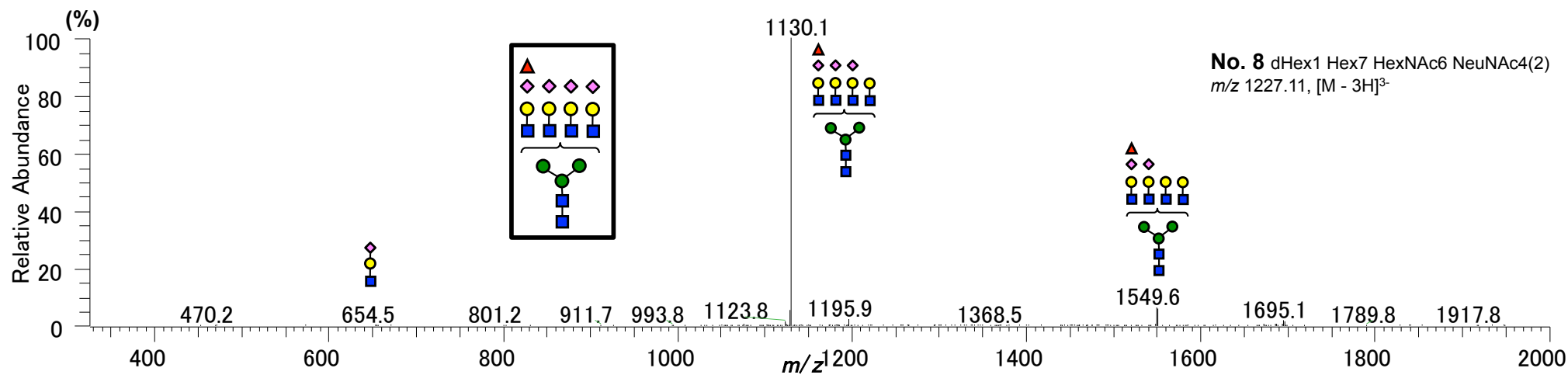

# Increased glycans

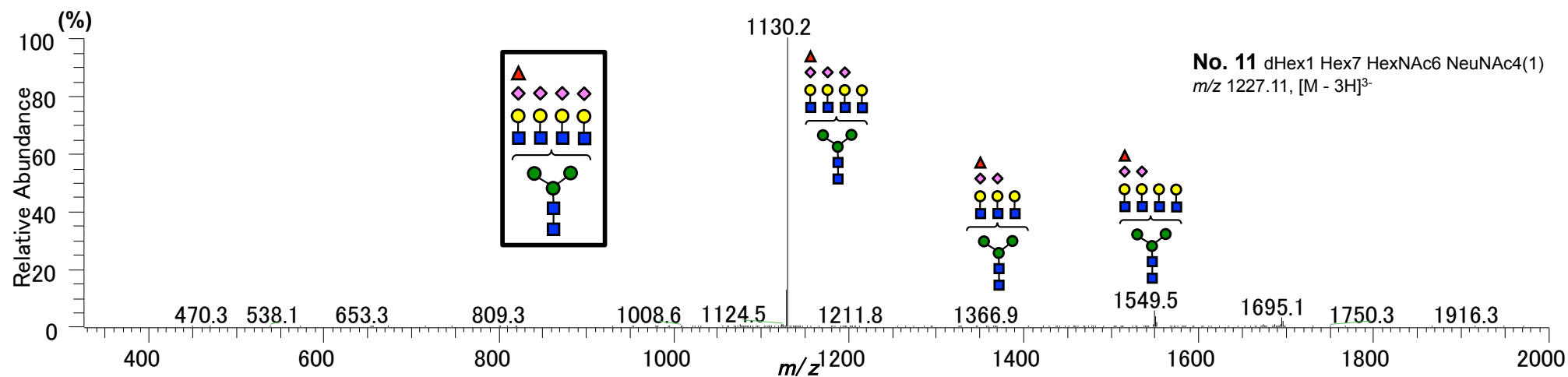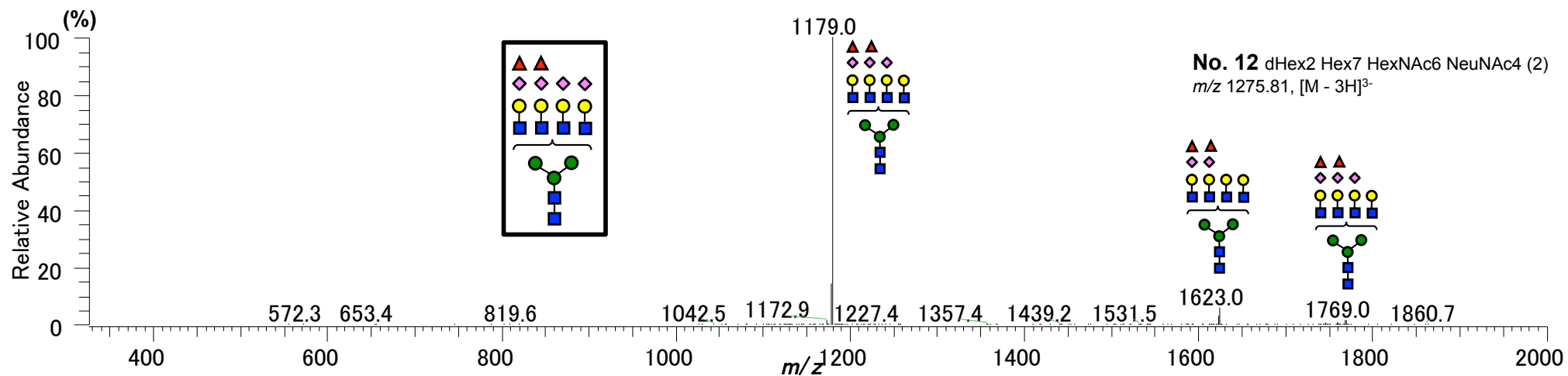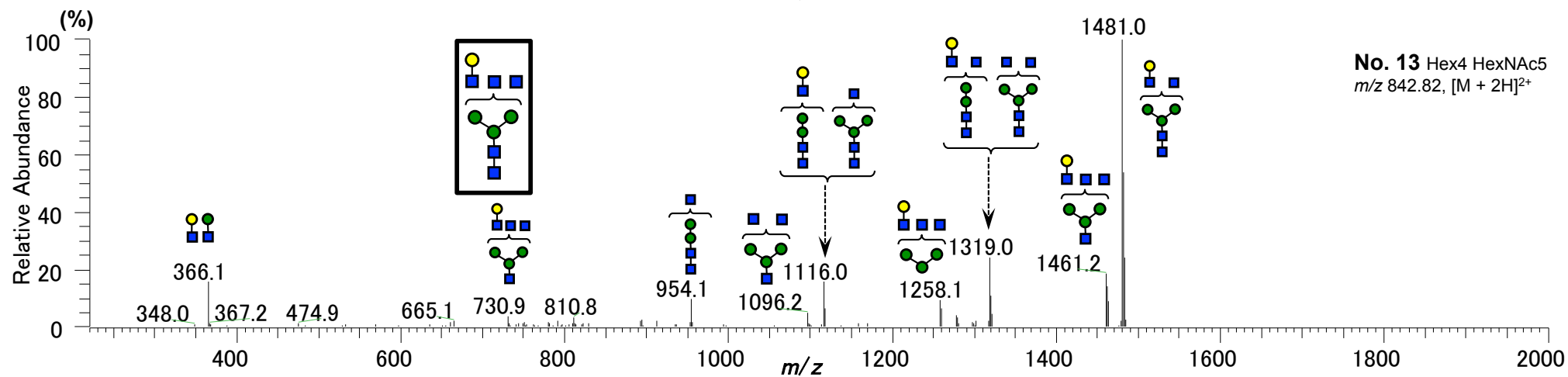

# Decreased glycans

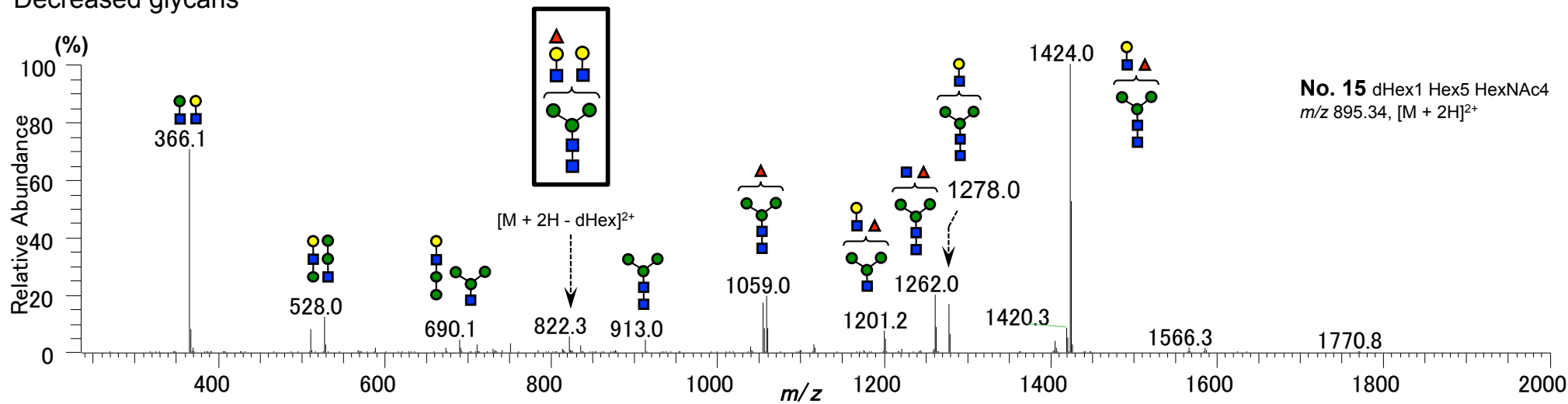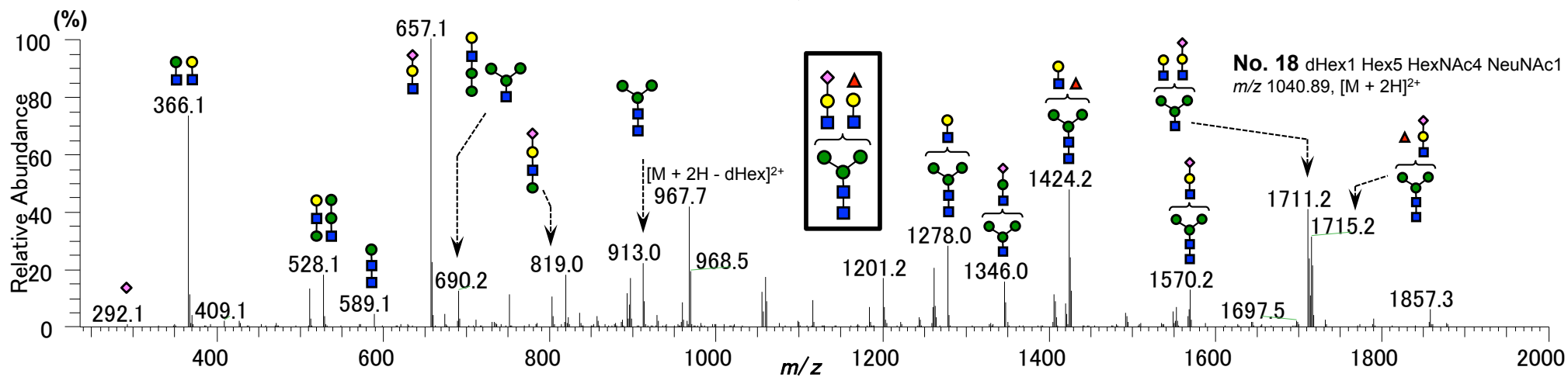

Supplement: S4 Fig — Spectra represent increased and decreased N-glycans in SSCs. N-glycans not shown here were deduced by each calculated exact mass. Numbers of the N-glycans correspond to Table 1. blue square, N-acetylglucosamine; yellow circle, galactose; green circle, mannose; purple diamond, N-acetylneuraminic acid; red triangle, fucose. (PDF) [file pone.0142645.s004.pdf]

S5 Fig.

(A)

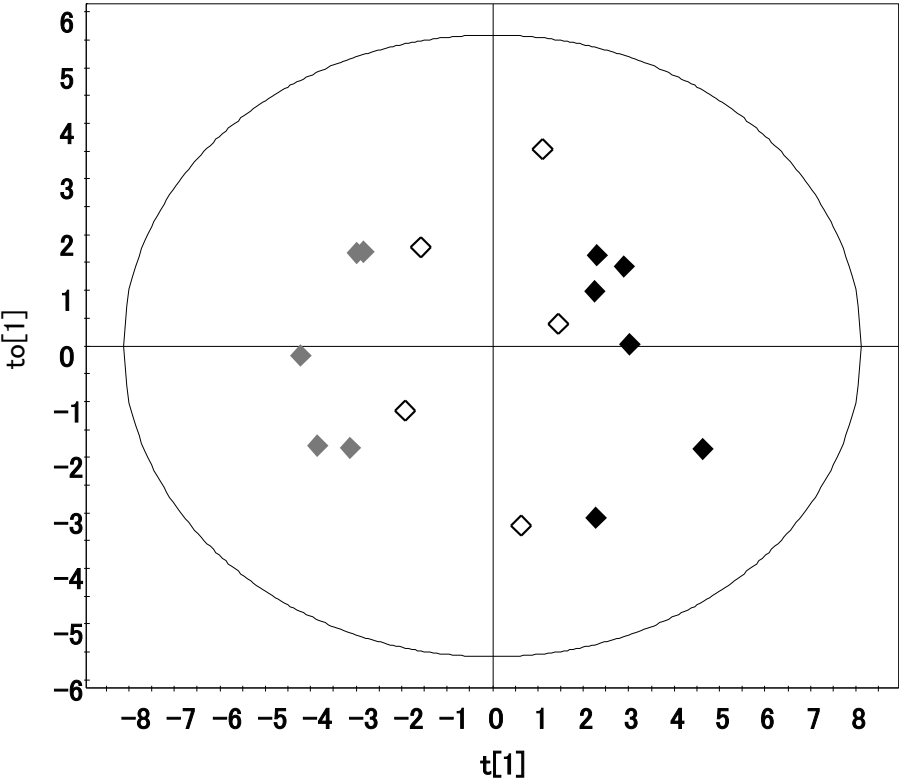

(B)

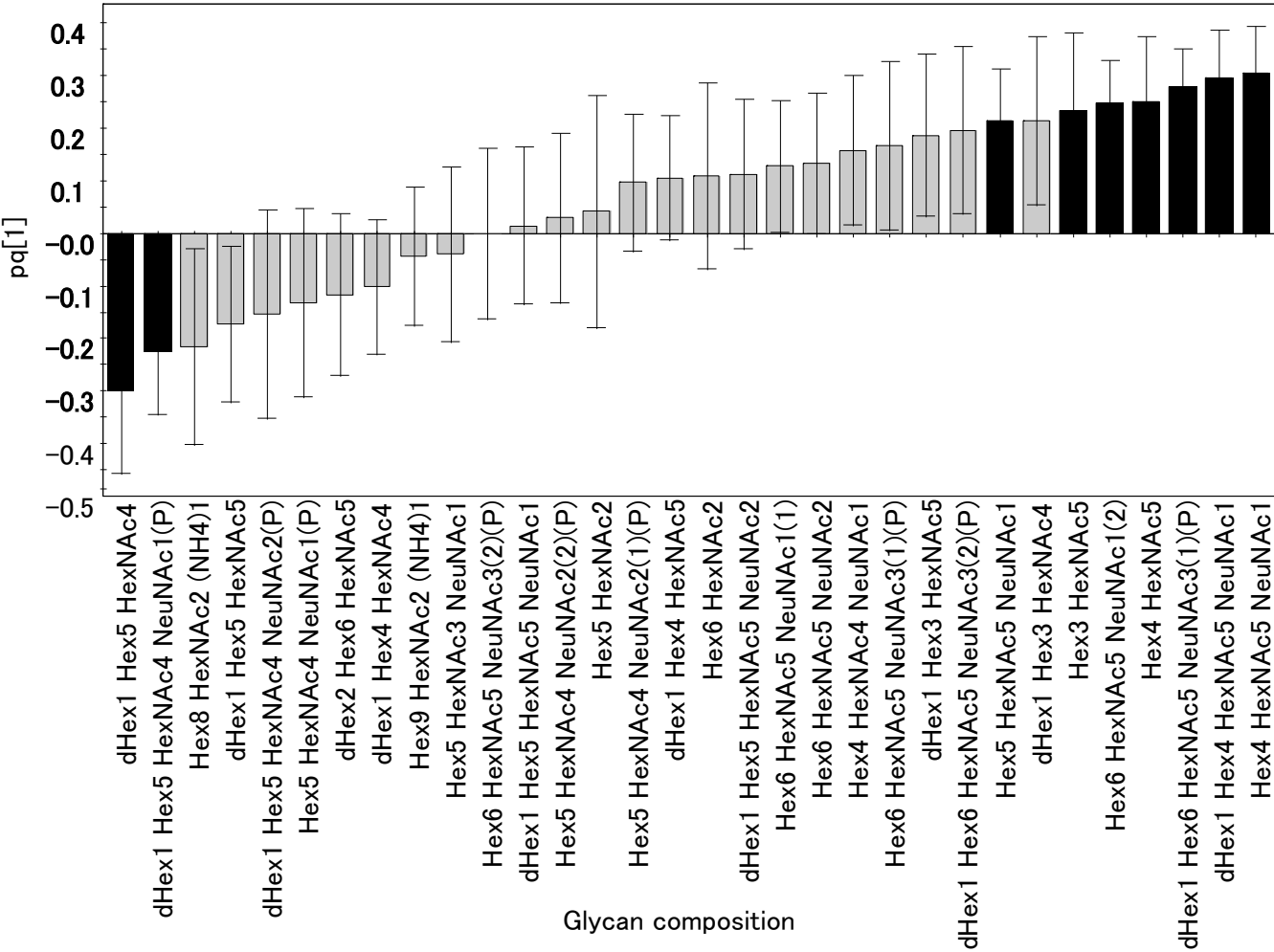

Supplement: S5 Fig — (A) An O-PLS score plot between young controls (shadowed), aged controls (opened), and SSCs (closed). R2X [1] = 0.263562, R2X [XSide Comp. 1] = 0.205859, Ellipse: Hotelling T2 (95%). (B) A loading plot by O-PLS between young controls, aged controls, and SSCs. Numbers in parentheses indicate isomers. "P" in parentheses indicates data obtained from the positive ion mode. Closed and shadowed columns represent [pq1] / SE > 1.5 and < 1.5, respectively. Hex, hexose; HexNAc, N-acetylhexosamine; NeuNAc, N-acetylneuraminic acid; dHex, deoxyhexose; NH4, ammonium. (PDF) [file pone.0142645.s005.pdf]

S6 Fig.

(A)

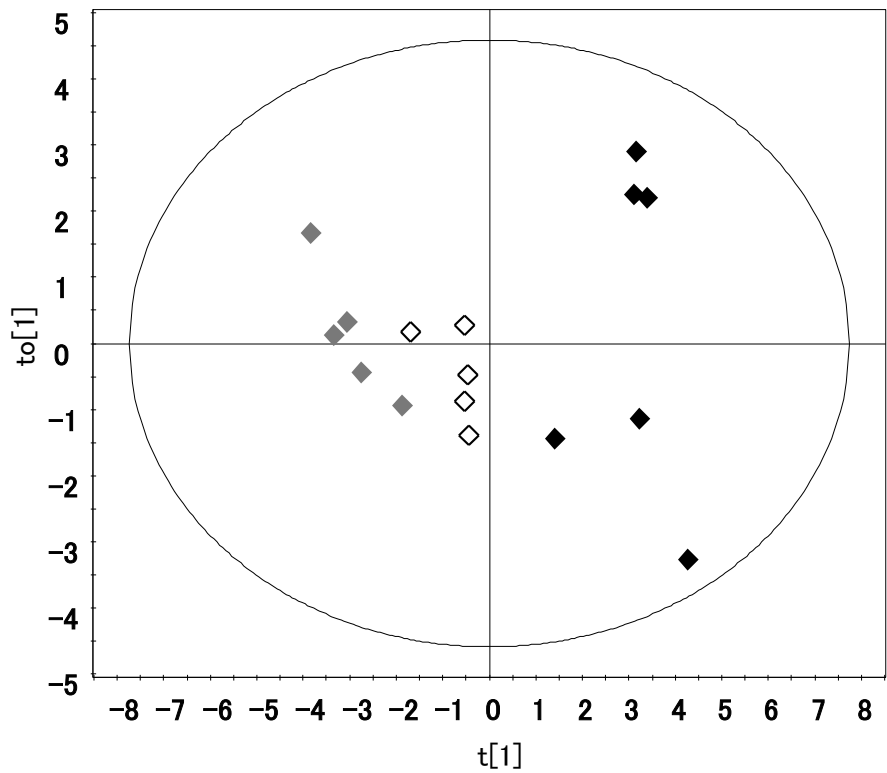

(B)

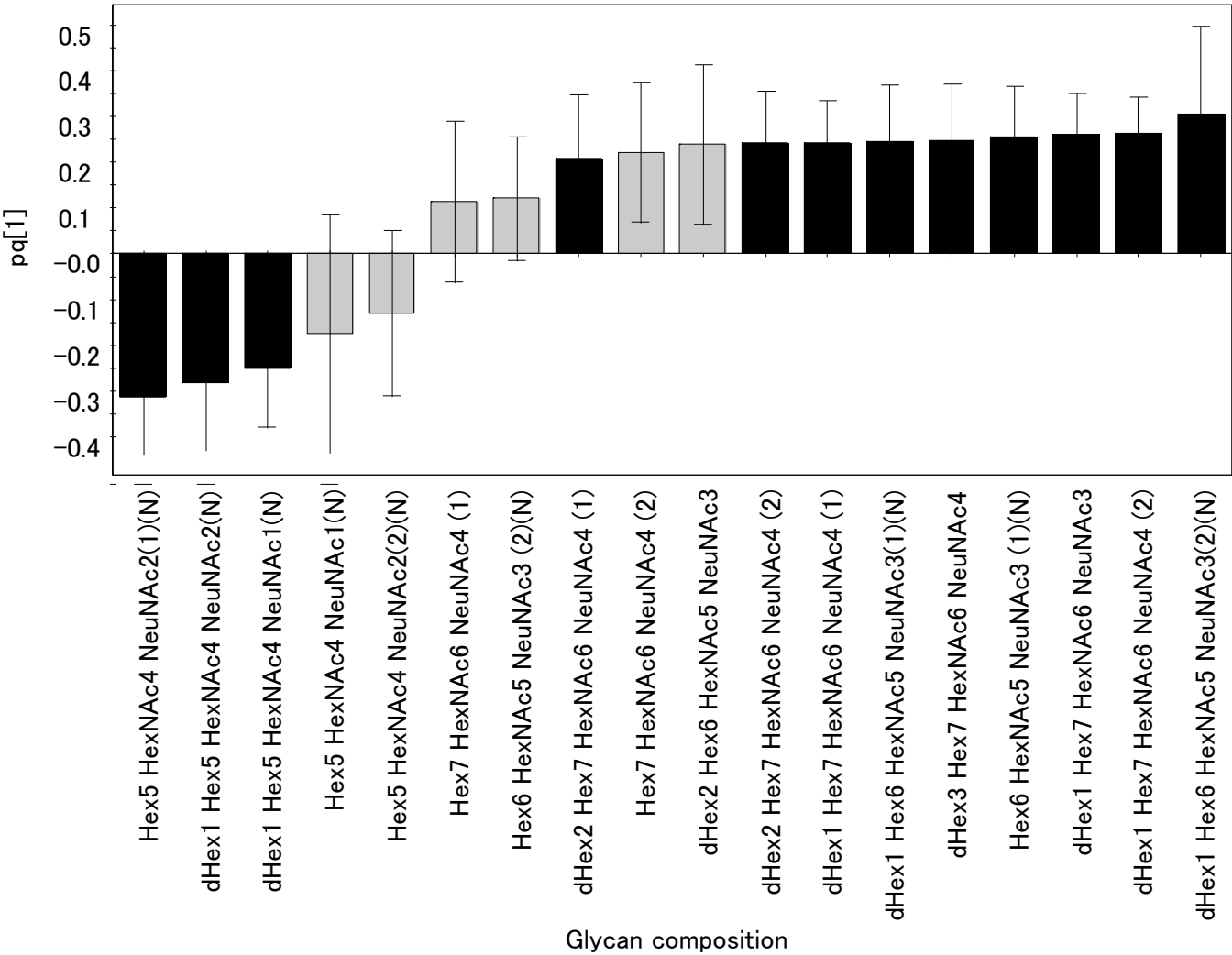

Supplement: S6 Fig — (A) An O-PLS score plot between young controls (shadowed), aged controls (opened), and SSCs (closed). R2X [1] = 0.416393, R2X [XSide Comp. 1] = 0.241192, Ellipse: Hotelling T2 (95%). (B) A loading plot by O-PLS between young controls, aged controls, and SSCs. Numbers in parentheses indicate isomers. "N" in parentheses indicates data obtained from the negative ion mode. Closed and shadowed columns represent [pq1] / SE > 1.5 and < 1.5, respectively. Hex, hexose; HexNAc, N-acetylhexosamine; NeuNAc, N-acetylneuraminic acid; dHex, deoxyhexose. (PDF) [file pone.0142645.s006.pdf]

**S7 Fig.**

**(A)**

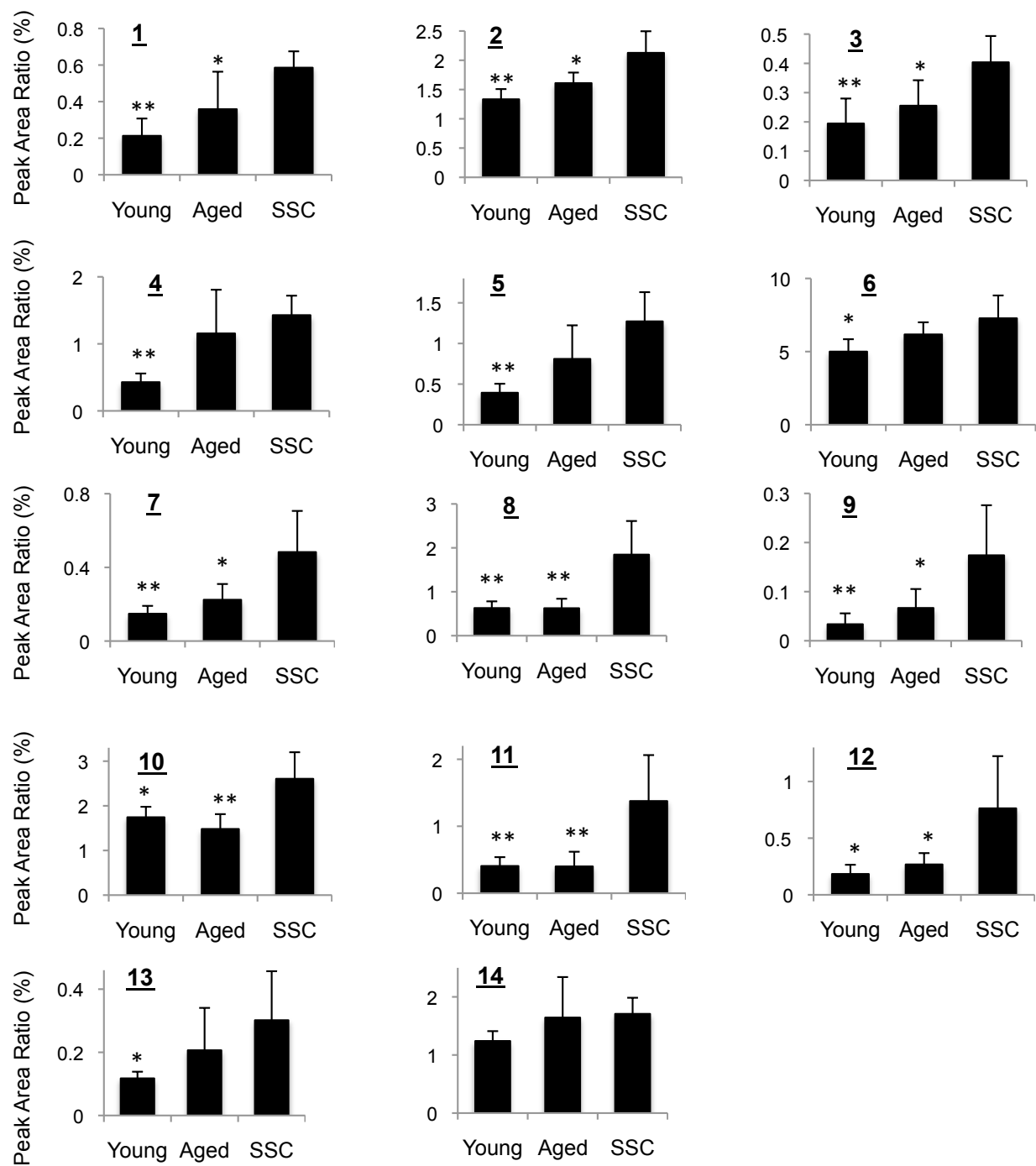

(B)

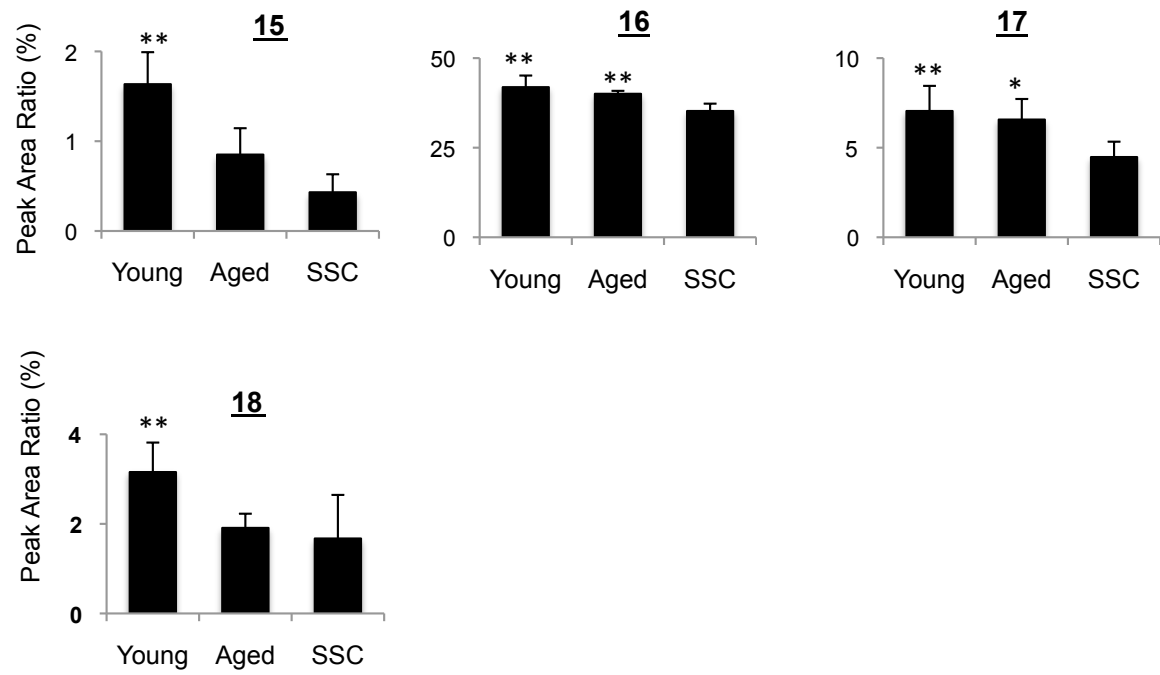

Supplement: S7 Fig — The numbers of graphs correspond to the glycan numbers in Table 1. The vertical axis indicates peak area ratios against the total peak area of all N-glycans in each ion mode. In the case of No. 2 and No.18, the data of the negative ion mode were used because the absolute pq[1] values in the negative mode were larger than that in the positive mode (shown in Fig 2C). Row data were summarized in S2 Table. Data represent the mean ± SD (n = 5 in young and aged, n = 6 in SSC). (A) Increased N-glycans in SSCs, (B) Decreased N-glycans in SSCs, *p < 0.05, **p < 0.01, vs SSC. (PDF) [file pone.0142645.s007.pdf]
