# Supplementary material for: Change in N-Glycosylation of Plasma Proteins in Japanese Semisupercentenarians
Source: PLoS One. 2015 Nov 11;10(11):e0142645. doi: 10.1371/journal.pone.0142645 (PMC4641608; doi:10.1371/journal.pone.0142645)
Supplement: S1 Table — 1Numbers in parentheses represent isomers, and "N" or "P" in parentheses indicates the data obtained from negative or positive ion mode, respectively. 2Hex, hexose; HexNAc, N-acetylhexosamine; NeuNAc, N-acetylneuraminic acid; dHex, deoxyhexose; NH4, ammonium. 3RT represents retention time. (PDF) [file pone.0142645.s008.pdf]

S1 Table. Retention time and peak area ratio of each *N*-glycan detected in positive and negative ion modes

				Young 1		Young 2		Young 3		Young 4		Young 5		Aged 1		Aged 2		Aged 3		Aged 4		Aged 5		SSC 1		SSC 2		SSC 3		SSC 4		SSC 5		SSC 6	
**Composition[1, 2]**	**Observed *m/z***	**Polarity**	**Charge state**	**RT[3]**	**Peak Area Ratio**	**RT**	**Peak Area Ratio**	**RT**	**Peak Area Ratio**	**RT**	**Peak Area Ratio**	**RT**	**Peak Area Ratio**	**RT**	**Peak Area Ratio**	**RT**	**Peak Area Ratio**	**RT**	**Peak Area Ratio**	**RT**	**Peak Area Ratio**	**RT**	**Peak Area Ratio**	**RT**	**Peak Area Ratio**	**RT**	**Peak Area Ratio**	**RT**	**Peak Area Ratio**	**RT**	**Peak Area Ratio**	**RT**	**Peak Area Ratio**	**RT**	**Peak Area Ratio**
**Hex5 HexNAc2**	**1237.47**	**Positive**	**1**	17.31	0.202	17.37	0.184	17.47	0.090	17.44	0.165	17.48	0.118	17.40	0.049	17.44	0.290	17.29	0.308	17.40	0.126	17.48	0.245	17.29	0.301	17.31	0.202	17.31	0.175	17.36	0.118	17.24	0.029	17.43	0.131
**Hex6 HexNAc2**	**1399.53**	**Positive**	**1**	14.49	0.069	14.65	0.083	14.66	0.055	14.70	0.057	14.68	0.059	14.63	0.030	14.70	0.097	14.58	0.069	14.59	0.100	14.64	0.042	14.63	0.165	14.62	0.067	14.69	0.059	14.61	0.044	14.6	0.118	14.76	0.076
**Hex8 HexNAc2 (NH_4)1**	**870.83**	**Positive**	**2**	13.72	0.632	13.89	0.475	14.08	0.432	13.91	0.309	13.88	0.311	13.96	0.350	13.89	0.264	13.79	0.523	13.80	0.417	13.86	0.421	13.82	0.340	13.88	0.289	13.95	0.378	13.86	0.196	13.85	0.150	13.98	0.166
**Hex9 HexNAc2 (NH_4)1**	**951.85**	**Positive**	**2**	13.85	1.451	14.15	1.096	14.22	1.099	14.18	1.299	14.01	1.508	14.09	0.581	14.16	0.928	13.92	1.533	14.04	2.022	14.13	2.468	14.04	0.773	13.99	1.271	14.07	1.456	13.98	1.003	14.08	1.037	14.13	0.855
**Hex3 HexNAc5**	**761.80**	**Positive**	**2**	10.11	0.036	10.12	0.053	10.11	0.046	10.25	0.059	10.23	0.022	10.40	0.081	10.24	0.095	10.13	0.190	10.12	0.080	10.13	0.491	10.38	0.087	10.13	0.325	10.27	0.252	10.41	0.092	10.27	0.688	10.27	0.122
**Hex4 HexNAc5**	**842.82**	**Positive**	**2**	11.34	0.140	11.38	0.129	11.35	0.117	11.49	0.118	11.48	0.084	11.66	0.112	11.50	0.130	11.25	0.187	11.36	0.145	11.45	0.415	11.51	0.171	11.43	0.385	11.39	0.279	11.55	0.178	11.43	0.574	11.57	0.223
**Hex5 HexNAc3 NeuNAc1**	**866.32**	**Positive**	**2**	14.23	0.223	14.52	0.278	14.66	0.439	14.31	0.218	14.15	0.262	14.09	0.071	14.28	0.395	14.19	0.257	14.17	0.366	14.24	0.255	14.04	0.075	14.37	0.494	14.2	0.270	13.98	0.276	14.08	0.200	14.25	0.213
**Hex4 HexNAc4 NeuNAc1**	**886.84**	**Positive**	**2**	15.24	0.488	15.37	0.560	15.56	0.647	15.36	0.680	15.17	0.325	15.11	0.360	15.34	0.789	15.19	1.441	15.26	0.955	15.24	1.254	15.01	0.464	15.24	1.099	15.03	0.693	14.95	0.820	14.98	0.947	15.19	0.804
**Hex5 HexNAc4 NeuNAc1(P)**	**967.86**	**Positive**	**2**	15.84	13.968	16.16	15.567	16.31	14.680	15.95	9.241	15.80	4.616	15.63	5.644	15.90	7.103	16.00	15.533	15.99	13.006	15.90	7.931	15.71	4.126	15.87	17.257	15.56	6.940	15.69	5.340	15.55	7.877	15.82	6.137
**Hex5 HexNAc4 NeuNAc2(1)(P)**	**1113.41(1)**	**Positive**	**2**	16.34	35.458	16.59	31.543	16.80	35.418	16.11	36.634	15.87	48.764	15.87	53.298	16.06	37.844	16.25	32.127	16.24	29.036	16.06	27.798	15.56	43.276	16.2	37.684	15.72	49.037	15.69	48.109	15.71	44.266	15.82	41.126
**Hex5 HexNAc4 NeuNAc2(2)(P)**	**1113.41(2)**	**Positive**	**2**	20.05	7.894	19.81	7.736	20.02	8.165	19.10	9.201	19.26	8.364	19.16	10.496	19.31	9.382	20.18	6.418	19.89	8.085	19.45	11.746	18.97	9.789	19.87	7.334	19.17	9.192	19.39	11.351	19.41	6.929	18.95	7.646
**Hex4 HexNAc5 NeuNAc1**	**988.37**	**Positive**	**2**	12.03	0.324	12.21	0.357	12.47	0.531	12.05	0.485	11.88	0.270	12.10	0.708	12.22	0.704	11.96	0.603	12.07	0.432	12.03	1.419	12.11	1.123	12.29	1.267	12.13	1.397	11.98	0.790	12.17	1.877	12.02	1.176
**Hex5 HexNAc5 NeuNAc1**	**1069.40**	**Positive**	**2**	12.86	1.215	13.19	1.310	13.34	1.366	13.04	1.355	12.88	0.957	12.97	0.715	13.21	2.561	12.96	1.496	12.92	1.426	12.88	1.718	12.98	1.920	13.14	1.635	13.13	1.699	12.85	1.363	13.02	2.124	13	1.502
**Hex6 HexNAc5 NeuNAc1(1)**	**1150.44(1)**	**Positive**	**2**	16.59	0.144	16.67	0.130	16.62	0.153	16.50	0.358	16.47	0.375	16.30	0.279	16.53	0.894	16.48	0.117	16.72	0.185	16.55	0.432	16.22	0.505	16.43	0.091	16.17	0.626	16.19	0.368	16.1	0.205	16.33	0.445
**Hex6 HexNAc5 NeuNAc1(2)**	**1150.44(2)**	**Positive**	**2**	17.96	0.093	18.16	0.314	18.14	0.146	17.83	0.240	17.94	0.179	17.77	0.120	17.99	0.344	18.10	0.251	18.11	0.305	18.07	0.216	17.68	0.393	17.86	0.278	17.56	0.444	17.81	0.461	17.62	0.326	17.66	0.520
**Hex6 HexNAc5 NeuNAc2**	**1295.99**	**Positive**	**2**	20.34	0.230	20.07	0.524	20.21	0.301	19.48	0.564	19.62	0.513	19.54	0.467	19.70	0.858	20.58	0.622	20.19	0.661	19.72	0.704	19.46	1.104	20.23	0.690	19.52	0.402	19.56	0.378	22.33	0.366	19.29	0.902
**Hex6 HexNAc5 NeuNAc3(1)(P)**	**961.35(1)**	**Positive**	**3**	19.85	0.658	19.66	0.928	19.84	0.627	18.78	2.096	18.82	1.794	18.81	2.111	18.92	2.384	19.92	1.219	19.71	2.383	19.15	3.561	18.47	1.697	19.7	0.869	18.73	2.280	18.83	3.055	18.98	2.256	18.44	2.105
**Hex6 HexNAc5 NeuNAc3(2)(P)**	**961.35(2)**	**Positive**	**3**	20.78	2.660	20.34	4.289	20.80	2.624	19.48	3.456	19.62	4.265	19.71	4.284	19.87	4.971	21.07	2.788	20.48	7.599	19.90	6.627	19.3	3.625	19.7	0.869	19.68	3.757	19.65	7.151	20.03	2.465	19.29	4.350
**dHex1 Hex3 HexNAc4**	**1465.59**	**Positive**	**1**	16.19	0.056	16.34	0.150	16.31	0.167	16.50	0.250	16.47	0.120	16.30	0.279	16.44	0.525	16.18	0.146	16.39	0.072	16.46	0.465	16.31	0.526	16.05	0.321	16.25	0.226	16.3	0.398	16.1	0.445	16.42	1.177
**dHex1 Hex4 HexNAc4**	**814.32**	**Positive**	**2**	17.09	2.311	17.17	2.903	17.18	2.187	17.25	3.251	17.25	1.793	17.08	1.557	17.14	2.676	17.11	3.669	17.21	5.664	17.19	4.465	17.09	0.743	16.89	1.846	16.93	1.105	17.15	1.027	16.84	2.268	17.06	1.664
**dHex1 Hex5 HexNAc4**	**895.34**	**Positive**	**2**	17.88	1.150	18.01	1.788	17.81	2.120	18.00	1.595	18.09	1.522	17.86	0.797	17.91	0.643	17.83	0.521	18.03	1.308	18.07	0.839	17.85	0.287	17.59	0.439	17.64	0.197	18.07	0.334	17.62	0.606	17.91	0.724
**dHex1 Hex3 HexNAc5**	**834.83**	**Positive**	**2**	12.86	0.287	13.05	0.258	13.03	0.215	13.35	0.399	13.19	0.215	13.27	0.372	13.21	0.329	12.82	1.756	13.07	0.404	13.14	1.650	13.25	0.283	13.01	1.085	13.13	0.586	13.14	0.413	13.15	1.124	13.28	1.006
**dHex1 Hex4 HexNAc5**	**915.86**	**Positive**	**2**	13.72	1.461	13.89	1.688	13.92	0.986	14.18	1.138	14.15	0.987	14.09	0.630	14.16	1.872	13.79	2.085	13.92	2.503	13.99	3.196	14.04	1.354	13.88	1.744	13.95	1.178	13.98	0.841	13.97	2.690	14.13	1.184
**dHex1 Hex5 HexNAc5**	**996.88**	**Positive**	**2**	14.23	1.477	14.41	1.693	14.50	1.016	14.58	0.757	14.56	0.842	14.50	0.626	14.57	0.506	14.32	0.835	14.31	1.289	14.50	0.655	14.42	0.602	14.37	1.112	14.45	0.683	14.36	0.427	14.35	1.077	14.65	0.583
**dHex2 Hex6 HexNAc5**	**1150.95**	**Positive**	**2**	14.72	6.930	15.01	5.964	15.16	3.678	14.95	2.563	14.80	1.733	14.75	0.209	14.96	1.649	14.81	6.337	14.72	4.537	14.88	4.855	14.63	1.382	14.73	4.064	14.69	2.884	14.61	1.009	14.6	2.698	14.76	1.490
**dHex1 Hex5 HexNAc4 NeuNAc1(P)**	**1040.89**	**Positive**	**2**	17.88	6.452	18.08	6.798	17.98	8.040	17.67	6.134	17.85	4.626	17.69	1.217	17.64	2.252	18.00	3.560	17.95	4.692	17.82	3.537	17.53	5.314	17.68	4.082	17.4	1.489	17.81	1.962	17.46	3.121	17.51	4.321
**dHex1 Hex5 HexNAc4 NeuNAc2(P)**	**1186.44**	**Positive**	**2**	18.23	6.434	18.35	6.249	18.41	8.780	17.75	10.527	17.77	9.002	17.61	10.563	17.74	9.038	18.37	4.450	18.20	7.018	17.90	6.110	17.38	7.394	18.11	6.130	17.48	5.069	17.65	5.808	17.54	6.316	17.43	8.073
**dHex1 Hex4 HexNAc5 NeuNAc1**	**1061.41**	**Positive**	**2**	13.97	0.559	14.26	0.393	14.50	0.283	14.18	0.567	14.15	0.340	14.09	0.484	14.28	1.187	14.19	1.046	14.17	0.747	14.13	2.072	13.93	1.926	14.25	1.349	14.2	1.239	13.86	1.150	14.08	1.617	14.13	1.290
**dHex1 Hex5 HexNAc5 NeuNAc1**	**1142.43**	**Positive**	**2**	14.72	6.032	15.01	5.307	15.29	3.756	14.95	4.240	14.80	4.038	14.75	1.143	14.83	5.024	14.81	8.795	14.84	3.455	14.88	2.974	14.63	6.837	14.85	4.011	14.79	3.734	14.61	3.252	14.6	3.541	14.76	5.667
**dHex1 Hex5 HexNAc5 NeuNAc2**	**1287.98**	**Positive**	**2**	15.60	0.824	15.86	1.082	16.13	1.557	15.36	1.661	15.17	1.600	15.11	1.976	15.45	3.392	15.68	0.752	15.63	0.726	15.36	1.214	14.92	2.716	15.68	1.169	15.24	1.403	14.95	1.538	15.09	1.377	15.1	3.477
**dHex1 Hex6 HexNAc5 NeuNAc3(1)(P)**	**1514.57(1)**	**Positive**	**2**	18.76	0.103	18.89	0.127	18.99	0.233	18.00	0.313	17.94	0.289	17.86	0.334	18.07	0.674	19.01	0.317	18.81	0.217	18.35	0.182	17.53	0.585	18.48	0.450	17.72	0.629	17.81	0.534	17.88	0.599	17.58	0.716
**dHex1 Hex6 HexNAc5 NeuNAc3(2)(P)**	**1514.57(2)**	**Positive**	**2**	22.18	0.036	21.47	0.042	22.16	0.045	20.91	0.071	20.77	0.108	21.05	0.058	21.05	0.198	20.91	0.047	20.92	0.040	21.07	0.044	20.71	0.116	20.62	0.093	21.29	0.240	20.89	0.215	21.65	0.088	20.63	0.127
**Hex5 HexNAc4 NeuNAc1(N)**	**965.85**	**Negative**	**2**	15.89	6.334	16.11	6.540	16.36	6.556	15.91	6.295	15.76	2.078	15.68	2.096	15.94	4.638	15.95	5.482	16.03	6.217	15.94	5.267	15.67	2.057	15.91	6.899	15.61	2.389	15.65	1.946	15.5	3.861	15.78	2.996
**Hex5 HexNAc4 NeuNAc2(1)(N)**	**1111.39(1)**	**Negative**	**2**	16.30	42.515	16.54	36.386	16.83	42.033	16.15	43.956	15.83	44.561	15.75	40.894	16.02	39.788	16.36	40.914	16.27	39.129	16.09	39.471	15.59	38.137	16.32	35.277	15.9	35.696	15.58	34.898	15.74	31.761	15.71	35.667
**Hex5 HexNAc4 NeuNAc2(2)(N)**	**1111.39(2)**	**Negative**	**2**	19.99	7.092	19.85	6.513	19.98	5.597	19.14	9.722	19.21	11.724	19.2	9.447	19.35	8.296	20.14	7.572	19.94	6.311	19.49	7.352	18.92	8.283	19.91	6.942	19.21	5.336	19.35	8.170	19.36	4.899	18.99	6.144
**Hex6 HexNAc5 NeuNAc3 (1)(N)**	**1439.52(1)**	**Negative**	**2**	19.81	5.250	19.61	6.051	19.88	4.012	18.72	4.262	18.76	5.421	18.75	5.155	18.95	6.013	19.86	7.039	19.66	5.664	19.1	6.992	18.42	5.879	19.64	4.849	18.77	8.600	18.87	8.088	19.02	8.616	18.48	7.622
**Hex6 HexNAc5 NeuNAc3 (2)(N)**	**1439.52(2)**	**Negative**	**2**	20.83	17.799	20.38	25.506	20.85	15.156	19.53	15.889	19.66	15.656	19.58	21.705	19.83	16.965	21.03	16.713	20.52	20.850	19.94	19.865	19.33	21.545	20.74	13.403	19.88	19.870	19.83	24.976	19.99	18.988	19.42	22.292
**Hex7 HexNAc6 NeuNAc4 (1)**	**1178.09(1)**	**Negative**	**3**	24.44	1.387	23.64	1.307	25.18	0.956	22.64	0.945	24.3	1.496	22.9	1.125	22.35	1.006	24.14	0.783	23.93	0.878	23.04	1.595	22.24	1.512	24.49	0.758	23.2	1.964	22.25	1.152	23.53	1.970	22.08	1.631
**Hex7 HexNAc6 NeuNAc4 (2)**	**1178.09(2)**	**Negative**	**3**	25.50	1.861	25	1.800	26.78	1.371	24.3	1.694	23.58	1.992	24.17	1.605	23.89	1.564	25.23	1.245	25.33	1.069	24.36	1.922	23.39	2.975	25.98	1.476	24.7	2.482	23.59	2.701	25.05	3.111	23.62	2.884
**dHex1 Hex5 HexNAc4 NeuNAc1(N)**	**1038.88**	**Negative**	**2**	17.84	3.207	18.05	3.730	18.02	3.868	17.71	2.435	17.74	2.543	17.56	1.732	17.69	1.863	17.96	1.521	18	2.335	17.76	2.098	17.57	3.353	17.73	1.637	17.43	0.568	17.69	0.918	17.4	1.564	17.54	2.000
**dHex1 Hex5 HexNAc4 NeuNAc2(N)**	**1184.43**	**Negative**	**2**	18.18	6.389	18.39	6.157	18.37	9.479	17.79	7.017	17.74	6.196	17.56	6.380	17.78	7.816	18.33	4.788	18.31	7.285	17.94	6.580	17.33	4.837	17.98	5.747	17.43	3.480	17.6	3.854	17.57	3.948	17.38	4.998
**dHex1 Hex6 HexNAc5 NeuNAc3(1)(N)**	**1512.55(1)**	**Negative**	**2**	18.90	5.104	18.92	3.763	18.94	7.589	18.03	4.659	17.89	5.011	17.82	6.202	18.11	7.690	18.96	10.263	18.76	6.697	18.3	5.490	17.57	5.939	18.52	13.386	17.68	9.805	17.85	8.009	17.92	11.455	17.54	7.516
**dHex1 Hex6 HexNAc5 NeuNAc3(2)(N)**	**1512.55(2)**	**Negative**	**2**	22.11	1.419	21.51	1.307	22.09	1.050	20.71	1.384	20.83	1.508	21.11	1.625	21.11	1.809	22.35	1.503	21.76	1.747	21.14	1.366	20.77	2.247	22.25	2.164	21.23	1.730	20.93	2.609	21.68	1.655	20.57	2.356
**dHex1 Hex7 HexNAc6 NeuNAc3**	**1695.12**	**Negative**	**2**	22.26	0.140	21.78	0.088	22.36	0.202	20.98	0.173	21.06	0.138	21.24	0.180	21.24	0.171	22.47	0.362	21.85	0.155	21.39	0.253	20.96	0.218	22.34	0.582	21.34	0.745	21.03	0.292	21.59	0.704	20.78	0.357
**dHex1 Hex7 HexNAc6 NeuNAc4 (1)**	**1226.77(1)**	**Negative**	**3**	23.03	0.376	22.36	0.198	23.47	0.533	21.22	0.419	21.06	0.504	21.51	0.515	21.24	0.543	22.09	0.043	22.45	0.335	21.73	0.565	20.96	0.920	23.02	1.304	21.71	2.402	21.13	0.644	22.1	2.006	20.78	0.982
**dHex1 Hex7 HexNAc6 NeuNAc4 (2)**	**1226.77(2)**	**Negative**	**3**	24.56	0.638	23.85	0.447	25.3	0.861	22.88	0.550	22.46	0.648	23	0.730	22.69	0.933	23.03	0.371	24.12	0.582	23.25	0.512	22.43	1.224	24.67	2.231	23.66	2.545	22.44	0.995	23.9	2.784	22.3	1.295
**dHex2 Hex6 HexNAc5 NeuNAc3**	**1585.58**	**Negative**	**2**	20.38	0.219	20.02	0.115	20.27	0.288	19.14	0.267	19.3	0.218	19.2	0.279	19.46	0.407	20.52	0.529	20.13	0.457	19.67	0.233	18.92	0.337	20.27	1.288	19.38	0.624	19.43	0.382	19.63	0.626	18.89	0.534
**dHex2 Hex7 HexNAc6 NeuNAc4 (1)**	**1275.47(1)**	**Negative**	**3**	20.29	0.085	20.02	0.023	20.4	0.111	18.83	0.074	18.87	0.066	18.87	0.076	19.05	0.133	20.43	0.317	20.03	0.092	19.4	0.136	18.33	0.104	20.18	0.549	19.03	0.432	18.87	0.067	19.28	0.585	18.39	0.142
**dHex2 Hex7 HexNAc6 NeuNAc4 (2)**	**1275.47(2)**	**Negative**	**3**	23.15	0.161	22.49	0.068	23.6	0.293	21.37	0.206	21.3	0.191	21.72	0.203	21.45	0.301	23.16	0.425	22.57	0.171	21.87	0.240	21.07	0.355	23.14	1.187	21.9	1.113	21.34	0.246	22.29	1.234	20.99	0.442
**dHex3 Hex7 HexNAc6 NeuNAc4**	**1324.14**	**Negative**	**3**	20.74	0.022		0.000	20.85	0.045	19.34	0.052	19.39	0.048	19.29	0.052	19.55	0.064	20.94	0.130	20.44	0.025	19.78	0.063	18.92	0.078	20.74	0.321	19.55	0.220	19.52	0.053	19.83	0.231	18.99	0.142

[1] Numbers in parentheses represent isomers, and "N" or "P" in parentheses indicates the data obtained from negative or positive ion mode, respectively.

[2] Hex, hexose; HexNAc, *N*-acetylhexosamine; NeuNAc, *N*-acetylneuraminic acid; dHex, deoxyhexose; NH4, ammonium.

[3] RT represents retention time.
