# Supplementary material for: Change in N-Glycosylation of Plasma Proteins in Japanese Semisupercentenarians
Source: PLoS One. 2015 Nov 11;10(11):e0142645. doi: 10.1371/journal.pone.0142645 (PMC4641608; doi:10.1371/journal.pone.0142645)
Supplement: S2 Table — 1Numbers in parentheses represent isomers, and "N" or "P" in parentheses indicates data obtained from the negative or positive ion mode, respectively. 2Hex, hexose; HexNAc, N-acetylhexosamine; NeuNAc, N-acetylneuraminic acid; dHex, deoxyhexose; NH4, ammonium. (PDF) [file pone.0142645.s009.pdf]

S2 Table. Weight (pq[1]) and its error (SE) of each *N*-glycan calculated using O-PLS

Var ID (Primary)[1, 2]	Var ID (Polarity)	Var ID (*m/z*)	pq[1]	SE	pq[1]/SE
**dHex1 Hex5 HexNAc4**	Positive	895.34	-0.2309	0.1004	2.2994
**Hex5 HexNAc4 NeuNAc2(1)(N)**	Negative	1111.39(1)	-0.2038	0.0893	2.2821
**dHex1 Hex5 HexNAc4 NeuNAc2(N)**	Negative	1184.43	-0.1908	0.1015	1.8791
**dHex1 Hex5 HexNAc4 NeuNAc1(N)**	Negative	1038.88	-0.1817	0.1001	1.8150
**dHex1 Hex5 HexNAc4 NeuNAc1(P)**	Positive	1040.89	-0.1765	0.0848	2.0820
**Hex8 HexNAc2 (NH4)1**	Positive	870.83	-0.1689	0.1412	1.1957
**dHex1 Hex5 HexNAc5**	Positive	996.88	-0.1398	0.1111	1.2589
**Hex5 HexNAc4 NeuNAc1(N)**	Negative	965.85	-0.1319	0.1412	0.9341
**dHex2 Hex6 HexNAc5**	Positive	1150.95	-0.1085	0.1422	0.7636
**Hex5 HexNAc4 NeuNAc1(P)**	Positive	967.86	-0.1078	0.1563	0.6894
**dHex1 Hex5 HexNAc4 NeuNAc2(P)**	Positive	1186.44	-0.1043	0.1444	0.7223
**dHex1 Hex4 HexNAc4**	Positive	814.32	-0.0843	0.0978	0.8619
**Hex5 HexNAc4 NeuNAc2(2)(N)**	Negative	1111.39(2)	-0.0733	0.1404	0.5222
**Hex9 HexNAc2 (NH4)1**	Positive	951.85	-0.0509	0.0922	0.5525
**Hex5 HexNAc3 NeuNAc1**	Positive	866.32	-0.0330	0.1343	0.2458
**dHex1 Hex5 HexNAc5 NeuNAc1**	Positive	1142.43	0.0087	0.1149	0.0761
**Hex6 HexNAc5 NeuNAc3(2)(P)**	Positive	961.35(2)	0.0300	0.1081	0.2772
**Hex5 HexNAc2**	Positive	1237.47	0.0324	0.1344	0.2410
**Hex5 HexNAc4 NeuNAc2(2)(P)**	Positive	1113.41(2)	0.0378	0.0867	0.4358
**dHex1 Hex4 HexNAc5**	Positive	915.86	0.0458	0.0969	0.4728
**Hex6 HexNAc2**	Positive	1399.53	0.0796	0.1267	0.6279
**Hex6 HexNAc5 NeuNAc3 (2)(N)**	Negative	1439.52(2)	0.0797	0.1084	0.7352
**Hex5 HexNAc4 NeuNAc2(1)(P)**	Positive	1113.41(1)	0.0848	0.1085	0.7818
**Hex7 HexNAc6 NeuNAc4 (1)**	Negative	1178.09(1)	0.0889	0.1232	0.7215
**dHex1 Hex5 HexNAc5 NeuNAc2**	Positive	1287.98	0.0991	0.1140	0.8695

Var ID (Primary)[1, 2]	Var ID (Polarity)	Var ID (*m/z*)	pq[1]	SE	pq[1]/SE
**Hex4 HexNAc4 NeuNAc1**	Positive	886.84	0.1007	0.1336	0.7534
**Hex6 HexNAc5 NeuNAc1(1)**	Positive	1150.44(1)	0.1026	0.0955	1.0747
**dHex1 Hex3 HexNAc5**	Positive	834.83	0.1104	0.1283	0.8610
**Hex6 HexNAc5 NeuNAc2**	Positive	1295.99	0.1177	0.0978	1.2032
**Hex3 HexNAc5**	Positive	761.8	0.1292	0.0898	1.4393
**Hex6 HexNAc5 NeuNAc3(1)(P)**	Positive	961.35(1)	0.1303	0.1192	1.0926
**dHex2 Hex7 HexNAc6 NeuNAc4 (1)**	Negative	1275.47(1)	0.1362	0.0915	1.4877
**Hex5 HexNAc5 NeuNAc1**	Positive	1069.4	0.1393	0.0668	2.0850
**Hex4 HexNAc5**	Positive	842.82	0.1466	0.0783	1.8736
**dHex2 Hex6 HexNAc5 NeuNAc3**	Negative	1585.58	0.1474	0.1331	1.1071
**dHex2 Hex7 HexNAc6 NeuNAc4 (2)**	Negative	1275.47(2)	0.1535	0.0818	1.8775
**dHex1 Hex6 HexNAc5 NeuNAc3(1)(N)**	Negative	1512.55(1)	0.1549	0.1151	1.3449
**dHex1 Hex7 HexNAc6 NeuNAc4 (1)**	Negative	1226.77(1)	0.1574	0.0789	1.9940
**Hex7 HexNAc6 NeuNAc4 (2)**	Negative	1178.09(2)	0.1608	0.0807	1.9926
**dHex3 Hex7 HexNAc6 NeuNAc4**	Negative	1324.14	0.1629	0.0960	1.6978
**dHex1 Hex7 HexNAc6 NeuNAc4 (2)**	Negative	1226.77(2)	0.1631	0.0718	2.2698
**dHex1 Hex6 HexNAc5 NeuNAc3(2)(P)**	Positive	1514.57(2)	0.1644	0.1104	1.4897
**dHex1 Hex3 HexNAc4**	Positive	1465.59	0.1674	0.1264	1.3240
**dHex1 Hex7 HexNAc6 NeuNAc3**	Negative	1695.12	0.1714	0.0712	2.4073
**Hex6 HexNAc5 NeuNAc3 (1)(N)**	Negative	1439.52(1)	0.1929	0.0592	3.2581
**Hex4 HexNAc5 NeuNAc1**	Positive	988.37	0.2006	0.0596	3.3625
**dHex1 Hex4 HexNAc5 NeuNAc1**	Positive	1061.41	0.2055	0.0683	3.0082
**Hex6 HexNAc5 NeuNAc1(2)**	Positive	1150.44(2)	0.2101	0.0368	5.7084
**dHex1 Hex6 HexNAc5 NeuNAc3(2)(N)**	Negative	1512.55(2)	0.2102	0.0921	2.2822
**dHex1 Hex6 HexNAc5 NeuNAc3(1)(P)**	Positive	1514.57(1)	0.2164	0.0570	3.7928

[1]Numbers in parentheses represent isomers, and "N" or "P" in parentheses indicates data obtained from the negative or positive ion mode, respectively.

[2] Hex, hexose; HexNAc, *N*-acetylhexosamine; NeuNAc, *N*-acetylneuraminic acid; dHex, deoxyhexose; NH4, ammonium.
